# Adaptive Evolution of *Mus Apobec3* Includes Retroviral Insertion and Positive Selection at Two Clusters of Residues Flanking the Substrate Groove

**DOI:** 10.1371/journal.ppat.1000974

**Published:** 2010-07-01

**Authors:** Bradley Sanville, Michael A. Dolan, Kurt Wollenberg, Yuhe Yan, Carrie Martin, Man Lung Yeung, Klaus Strebel, Alicia Buckler-White, Christine A. Kozak

**Affiliations:** 1 Laboratory of Molecular Microbiology, National Institute of Allergy and Infectious Diseases, Bethesda, Maryland, United States of America; 2 Bioinformatics and Computational Biosciences Branch, Office of Cyber Infrastructure and Computational Biology, National Institute of Allergy and Infectious Diseases, Bethesda, Maryland, United States of America; Harvard Medical School, United States of America

## Abstract

Mouse *APOBEC3* (mA3) is a cytidine deaminase with antiviral activity. mA3 is linked to the *Rfv3* virus resistance factor, a gene responsible for recovery from infection by Friend murine leukemia virus, and mA3 allelic variants differ in their ability to restrict mouse mammary tumor virus. We sequenced mA3 genes from 38 inbred strains and wild mouse species, and compared the mouse sequence and predicted structure with human *APOBEC3G* (hA3G). An inserted sequence was identified in the virus restrictive C57BL strain allele that disrupts a splice donor site. This insertion represents the long terminal repeat of the xenotropic mouse gammaretrovirus, and was acquired in Eurasian mice that harbor xenotropic retrovirus. This viral regulatory sequence does not alter splicing but is associated with elevated mA3 expression levels in spleens of laboratory and wild-derived mice. Analysis of *Mus* mA3 coding sequences produced evidence of positive selection and identified 10 codons with very high posterior probabilities of having evolved under positive selection. Six of these codons lie in two clusters in the N-terminal catalytically active cytidine deaminase domain (CDA), and 5 of those 6 codons are polymorphic in *Rfv3* virus restrictive and nonrestrictive mice and align with hA3G CDA codons that are critical for deaminase activity. Homology models of mA3 indicate that the two selected codon clusters specify residues that are opposite each other along the predicted CDA active site groove, and that one cluster corresponds to an hAPOBEC substrate recognition loop. Substitutions at these clustered mA3 codons alter antiviral activity. This analysis suggests that mA3 has been under positive selection throughout *Mus* evolution, and identified an inserted retroviral regulatory sequence associated with enhanced expression in virus resistant mice and specific residues that modulate antiviral activity.

## Author Summary

*APOBEC3* (mA3) is a cytidine deaminase with antiretroviral activity. Genetic variants of mA3 are associated with the restriction factor *Rfv3* (recovery from Friend leukemia virus) and with resistance to mouse mammary tumor virus. We sequenced mA3 from laboratory strains and wild mouse species to examine its evolution. We discovered that the mA3 allele in virus resistant mice is disrupted by insertion of the regulatory sequences of a mouse leukemia virus, and this insertion is associated with enhanced mA3 expression. We also subjected the *Mus* mA3 protein coding sequences to statistical analysis to determine if specific sites are subject to strong positive selection, that is, show an increased number of amino acid replacement mutations. We identified 10 such sites, most of which distinguish the mA3 genes of *Rfv3* virus restrictive and nonrestrictive mice. Six of these sites are in two clusters that, in human *APOBEC3G*, are important for function. We generated a structural model of mA3, positioned these clusters opposite each other along the putative mA3 active site groove, and demonstrated that substitutions at these sites alter antiviral activity. Thus, mA3 has been involved in genetic conflicts throughout mouse evolution, and we identify an inserted regulatory sequence and two codon clusters that contribute to mA3 antiviral function.

## Introduction

Species susceptible to infectious retroviruses have evolved numerous constitutively expressed antiviral factors that target various stages of the retroviral life cycle. The factors responsible for this intrinsic immunity include 3 that act at post-entry stages of virus replication: *Fv1*, *APOBEC3* and *TRIM5α*. *Fv1* was discovered in mice, [1] and only mice carry *Fv1* [2], [3]. *TRIM5α* was initially identified in primates as an anti-HIV-1 restriction factor [4], [5], and while mice carry *TRIM5α* related sequences [6], no mouse orthologue with virus restriction activity has been identified. Active *APOBEC3* genes, on the other hand, are found in various species including human and mouse, and mouse and human *APOBEC3* have antiviral activity against multiple retroviruses [reviewed in 7].

The APOBEC3 editing enzyme is incorporated into budding virions. During reverse transcription in subsequently infected cells, the virion-associated APOBEC3 catalyzes C-to-U deamination, resulting in G-to-A mutations in the viral DNA [8]. The increased mutational load has a major impact on viral fitness, and there is also some evidence that APOBEC3 antiviral activity is enhanced by additional deamination-independent mechanisms that act before proviral integration [9], [10].

APOBEC3 was initially described in primates, and human *APOBEC3* paralogues responsible for resistance are present as a cluster of 7 genes on chromosome 22, the most extensively studied of which is *APOBEC3G* (hA3G). HIV-1 can avoid inhibition by hA3G through the action of one of its viral accessory proteins, Vif (viral infectivity factor), that prevents incorporation of hA3G into the virion [11]. The antiviral activity of hA3G can be observed with Vif-negative HIV-1 and SIV lentiviruses as well as other retroviruses such as equine infectious anemia virus (EIAV) and mouse leukemia viruses (MLVs). In the mouse, there is only a single *APOBEC3* copy (mA3) on chromosome 15. Several observations indicate that mA3 functions in antiviral defense: mA3 inhibits infection by several viruses including HIV-1 and mouse retroviruses such as mouse mammary tumor virus (MMTV), intracisternal A-particles (IAPs) and MusD endogenous retroviruses [12]–[14]; mA3 knockout mice are more susceptible to MMTV infection and tumorigenesis [15]; endogenous retroviruses (ERVs) of MLV in the sequenced *Mus* genome show modifications consistent with APOBEC3 activity [16].

Two recent studies proposed that mA3 is responsible for the Friend virus resistance factor *Rfv3* [10], [17]. *Rfv3* is one of several host resistance factors that, like *Fv1*, were discovered in studies with the pathogenic Friend MLV (FrMLV) [18]. *Rfv3* was identified as a non-major histocompatibility complex gene that influences the duration of viremia, partly through its effects on the production of virus-neutralizing antibodies [19]. The prototype *Rfv3* virus restrictive strain is C57BL, and BALB/c is the prototype non-restrictive strain. The *Rfv3* gene map location on chromosome 15 [20] has now been linked to the locus of *Apobec3* [10], [17]. That mA3 is responsible for *Rfv3* resistance is supported by the observations that mA3 of C57BL restricts FrMLV replication and FrMLV-induced disease more effectively than BALB/c mA3, and that genetic inactivation of mA3 generates an FrMLV susceptible phenotype [10], [17]. It has also been shown that the C57BL mA3 allelic variant is more effective than the BALB/c allele in restricting MMTV [21].

The mA3 genes in prototype *Rfv3* restrictive and nonrestrictive strains differ in protein sequence, splicing pattern, and expression level, and all three of these factors may contribute to resistance [10], [17], [21]. Few strains and *Mus* species have been characterized for these differences [21], so we sequenced mA3 genes from various inbred strains and wild mice representative of the major taxonomic groups of *Mus*. In this paper, we demonstrate that an MLV long terminal repeat (LTR) disrupts a splice donor site in the mA3 of C57BL and other strains and species and is associated with altered expression levels, we demonstrate strong positive selection of this gene in *Mus* that involves sites that distinguish the mA3 genes of *Rfv3* virus resistant and susceptible mice, we use homology modeling to position the positively selected residues in two clusters on opposite sides of the putative active site groove, and we describe the antiviral activity of mA3 genes carrying mutations at these sites.

## Results

### Analysis of *Mus musculus* subspecies and inbred strains for *Apobec3* variants

**Figure 1:**
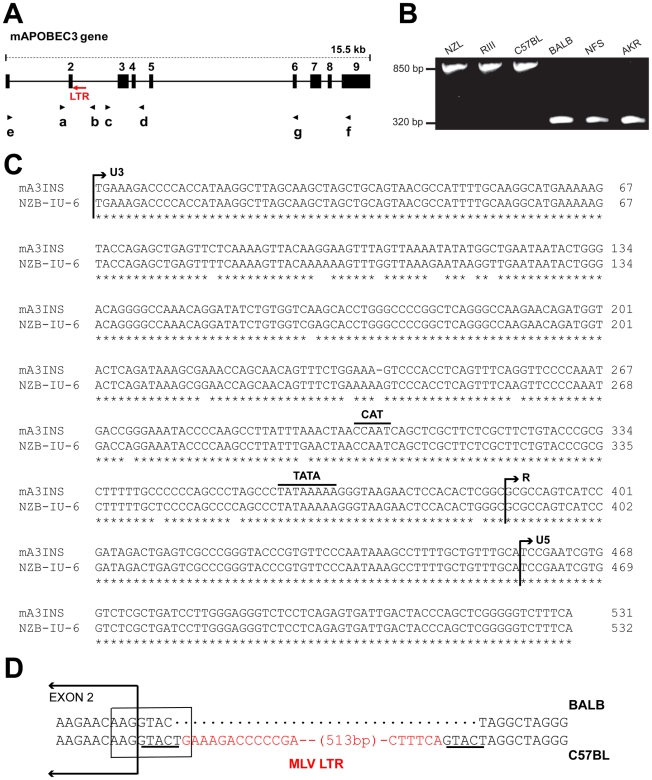
Genomic structure of mA3. A) Schematic representation of mA3. The nine exons are indicated by black boxes and the black arrows indicate the positions of the 7 PCR primers. The red arrow shows the position of the MLV LTR. B) PCR products of the indicated strains following amplification with primers a and b. C) The sequences of the 531 bp mA3 C57BL intron insertion and the LTR of the NZB-IU-6 X-MLV (GenBank No. K02730) were aligned using ClustalW [62] and marked to indicate the positions of the boundaries of the U3, R, and U5 regions and the CAT and TATA boxes. Asterisks indicate identical bases in the two sequences. D) Position of the inserted LTR in mA3. The insertion is present in C57BL and absent from BALB/c mice. A box encloses the splice donor site at the end of exon 2. The direct repeats flanking the LTR insertion are underlined and the LTR sequence is in red.

Analysis of the antiviral activities of chimeric and wild type C57BL and BALB/c mA3s by Takeda and colleagues [10] indicated that the mA3 anti-FrMLV activity resides in the N-terminal half of the C57BL protein. This 194 amino acid residue segment contains the active Z2-type cytidine deaminase region (CDA) [22], [23], and the translated protein sequences of restrictive C57BL and nonrestrictive BALB/c prototypes differ from one another in this region at nine residues [10]. To determine the distribution of the restrictive variant among mice and to identify novel variants, we sequenced segments of mA3 containing these 9 residues from inbred strains and wild-derived mice representing different taxa and/or mice trapped in different geographic locations (Table S1)(Figure 1A).

In the course of this analysis, we identified a 531 bp sequence inserted into the intron of mA3 of some laboratory strains between exons 2 and 3 (Figure 1A,1B). The insertion was sequenced and identified as an intact retroviral LTR (Figure 1C). This LTR is 96.6% identical to the LTR of the xenotropic gammaretrovirus (X-MLV) NZB-IU-6, an MLV isolated from NZB strain mice [24], [25]. The mA3 LTR insert shows the expected direct repeats characteristic of retroviral insertions, CAT and TATA boxes, and a comparable enhancer region. The LTR is inserted in an antisense orientation, and the site of insertion is the splice donor site at the end of exon 2 (Figure 1D). Part of the splice donor site contributes to the direct repeat flanking the insertion. The insertion alters the last base of the splice donor site, a position that is not highly constrained in the consensus sequence.

**Figure 2:**
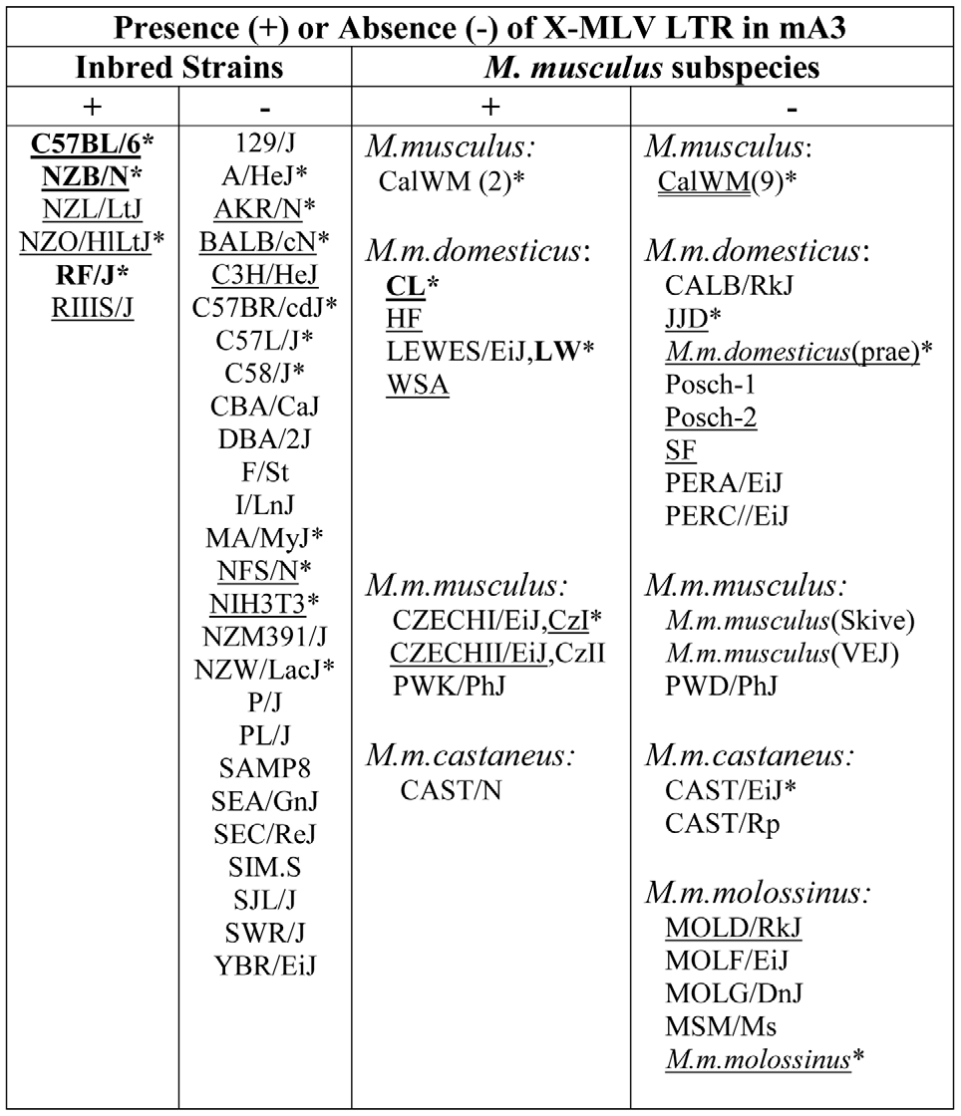
Distribution of the X-MLV LTR in inbred strains and wild-derived *M. musculus* house mouse species. An asterisk marks samples that were sequenced, and those examined for splice variants are underlined. Mice found to carry the virus restrictive C57BL mA3 sequence are bolded. CZECHI/CzI, CZECHII/CzII and LEWES/LW mice were sampled twice, from DNAs extracted from mice obtained from M. Potter in 1984, and from The Jackson Laboratory DNAs isolated from inbred lines developed from the same colonies. *M. musculus* mice trapped in California (CalWM) are listed without subspecies designation. Numbers in parentheses are the number of individuals tested. These 11 CalWM include mice trapped in Bouquet Canyon, Lake Casitas, and the SC-1 cell line.

We screened 32 laboratory mouse strains for presence of this LTR insertion by PCR (Figure 2). The insertion was identified in 6 strains, including C57BL and the 3 related strains NZB, NZL and NZO. The LTR was absent from other NZB-related strains, from other strains in the C57/C58 series and was also absent from 21 strains from other families of inbred strains. The sequences of exons 2–4 of 13 strains were compared, and the only strains identified as having the C57BL/6 coding sequence, NZB and RF, also carried the LTR insertion. (Figure 2).

The common inbred strains of mice are a mosaic of Eastern European *M. m. musculus*, Western European *M. m. domesticus* and Asian *M. m. castaneus* [26], [27]. Therefore we looked for the sequence polymorphisms associated with the C57BL allele and for the MLV LTR in *M. musculus* subspecies from breeding stocks established from mice trapped in Old World sites where these commensal (house mouse) subspecies originated, and from *M. musculus* mice trapped in the Americas where they had been introduced from Europe and Asia (Figure 2). Two wild-derived mice from the Delmarva (Delaware-Maryland-Virginia) Peninsula, CL and LEWES, had this LTR along with the C57BL mA3 coding sequence. PCR fragments diagnostic of the LTR insert were also found in other Maryland mice as well as in two mice trapped in California, one of three *M. m. castaneus* breeding lines, and three of four lines developed from mice trapped in the former Czechoslovakia. The LTRs sequenced in 4 laboratory strains and 5 wild-derived mice were 99% identical to one another, and the mA3 genes of the LTR+ wild mice had several substitutions compared to the C57BL gene. Thus, the LTR was acquired in Eurasian species, and these LTR modified mA3 genes continued to accumulate mutations after this insertion event.

**Figure 3:**
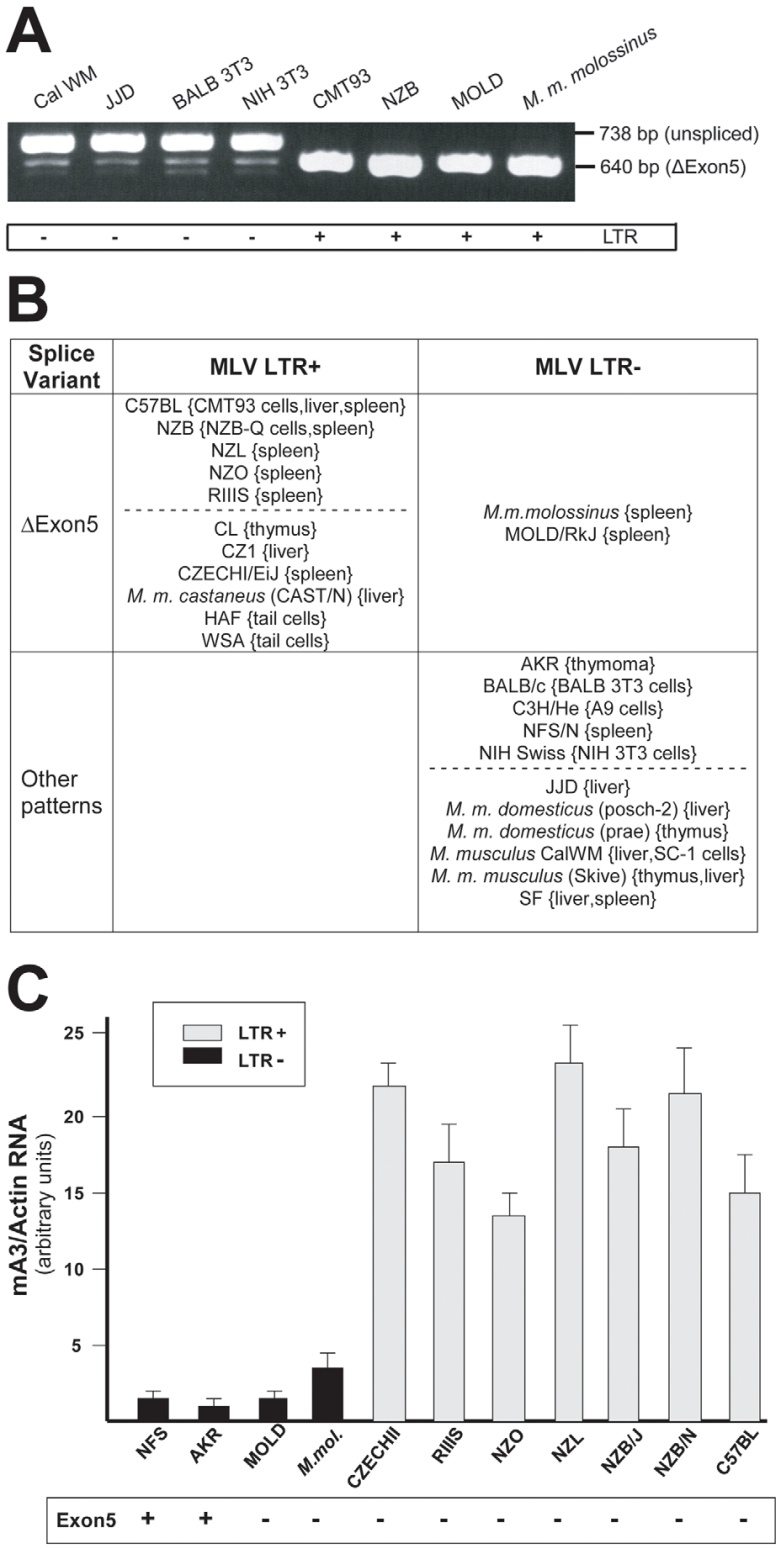
mA3 splicing patterns and expression levels in mouse strains and *M. musculus* subspecies. A) RT-PCR products from the indicated sources using primers a and g. B) Splicing patterns observed in mRNAs of mice carrying LTR+ and LTR− mA3 alleles. Mice listed as having “other patterns” produce the exon5+ isoform with one or more smaller mRNAs. The dotted lines separate common laboratory strains and wild-derived mice. The tissue or cell source of RNA is indicated in {}. C) Quantitative real time PCR of mA3 transcripts in spleens of LTR+ and LTR− laboratory and wild-derived mouse strains. Amplification levels were normalized to β-actin. The mice producing the exon5+ isoform are indicated at the bottom. Sequence analysis of NFS/N and AKR mA3 indicates they carry the BALB/c allele.

Previous reports had determined that mA3 mRNAs can lack exon 5 [10], [13], [14], and that BALB/c mA3 can also lack exon 2 [10]. We examined 31 mA3 mRNAs from cultured cells or tissues of 24 different inbred strains and wild-derived *M. musculus* mice for these splice variants by RT-PCR (Figure 3). mA3 mRNAs isolated from different tissues of the same mouse produced the same pattern of PCR products. Eleven of these 24 mice carry the LTR (Figure 3B), and all 11 mice produced a single PCR product of the size expected for a spliced message lacking exon 5 (Figure 3A). Among the 13 LTR-free mice, two, *M. m. molossinus* and the LTR− inbred MOLD/RkJ line of this subspecies, produced this same single isoform, while the other 11 LTR− mice additionally produced an exon5+ message that in 10 mice was significantly more abundant than the Δexon5 isoform (Figure 3A). Both sequenced BALB 3T3 mA3s lacked exons 2 and 5, and a third barely detectable smaller PCR product was observed in BALB 3T3 and other LTR− mice of the size consistent with the absence of exons 2 and 5 (Figure 3A). The distribution of the MLV LTR among these mice suggests that the LTR was inserted into the mA3 variant that produces the Δexon5 isoform.

Previous reports had noted that mA3 expression level is significantly higher in C57BL mouse tissues (LTR+) than in BALB/c (LTR−) [10], [21]. We isolated total RNA from the spleens of 11 mice that had been typed for the LTR and for mA3 splicing patterns. Included were mice from 2 breeding lines of *M. m. molossinus*, the inbred MOLD/RkJ strain and a mouse from a random bred colony, both of which are LTR− and produce the Δexon5 isoform (Figure 3B). Quantitative real-time PCR analysis showed that the 7 LTR+ mice produced 4–20 fold higher levels of mA3 mRNA than did the 4 LTR− mice, including the two *M. m. molossinus* mice (Figure 3C). These data demonstrate a correlation between the LTR and expression level but not splicing pattern.

### Selection analysis of mA3

We used sequenced segments of mA3 from 4 inbred strains and 21 wild-derived mouse species and subspecies for phylogenetic analysis. The sequences were used to construct phylogenies, and were analyzed with the PAML suite of programs [28] for evidence of adaptive evolution and to identify possible sites of positive selection. Two sets of DNA sequences were analyzed separately: exons 2–4 amplified from genomic DNA or RNA and a set of 8 near full length DNAs generated by RT-PCR (Text S1, S2). The sequences in the smaller dataset of 8 DNAs do not include the extreme 5′ and 3′ends of the gene or exon 5 which was absent from all but 3 of the 8 sequenced mRNAs.

**Figure 4:**
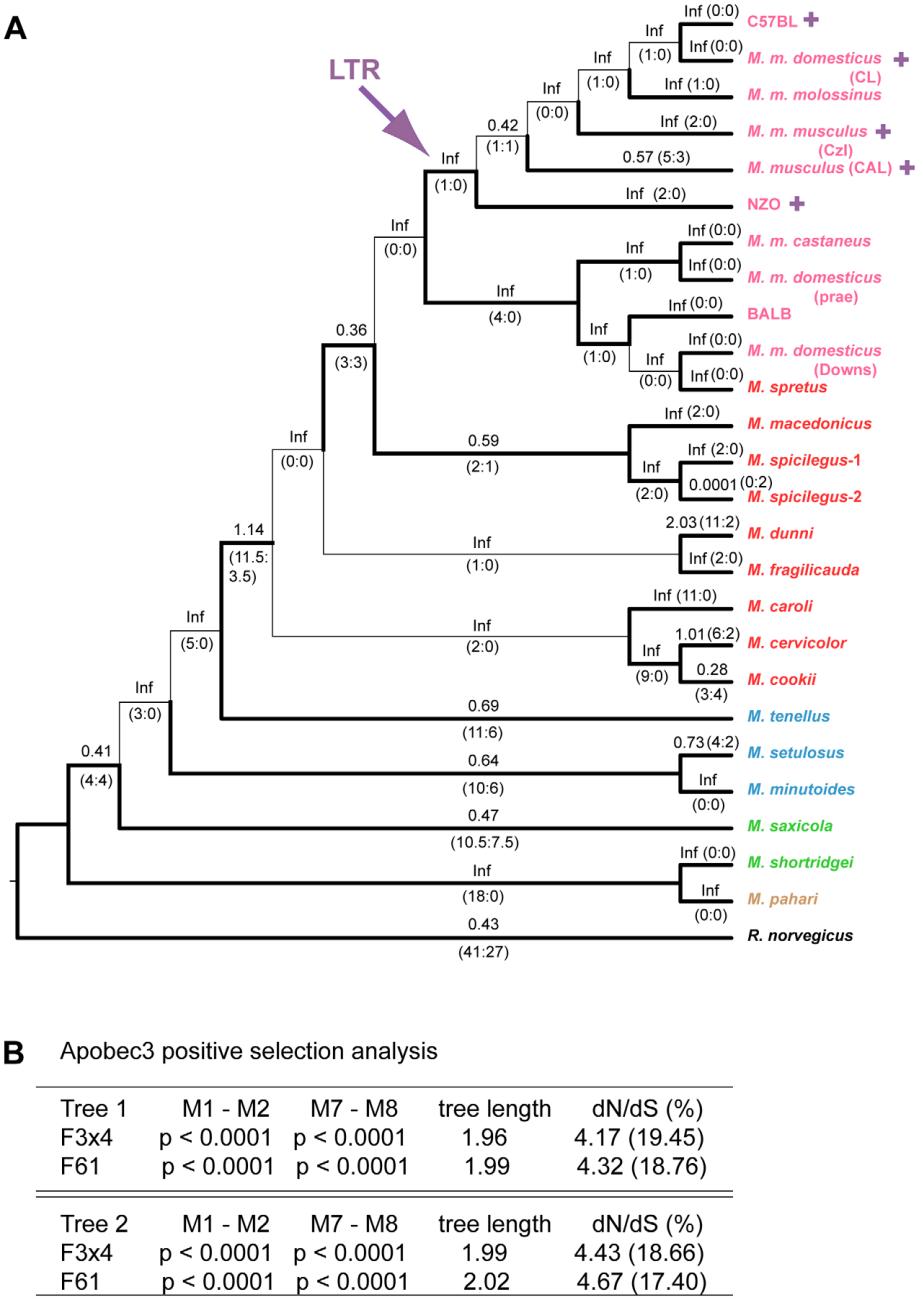
Positive selection on exons 2–4 of mA3 in *Mus*. A) Cladogram showing branch values of dN/dS calculated using the free-ratio model of PAML, with the number of replacement and synonymous changes in parentheses. When dS = 0, dN/dS is infinite (Inf). dN/dS>1 suggests positive selection along that lineage. Bootstrap support for this tree topology was generally good, with most bootstrap percentages >90%. The thin lines represent branches with bootstrap values <70%. Colors indicate *Mus* subgenera: brown, *Coelomys*; green, *Pyromys*; blue, *Nannomys*; red, *Mus* with *Mus* house mouse (*M. musculus*) strains and species in pink. A purple arrow indicates insertion of the X-MLV LTR, and purple plus signs identify taxa carrying the LTR. B) Likelihood ratio tests were used to test for positive selection. Neutral models (M1, M7) were compared with selection models (M2, M8) using two different models of codon frequency (F3X4 or F61). P values <0.0001 provide strong evidence of positive selection. Tree 1 is the data-derived tree and tree 2 is the taxonomy-derived tree. Tree length is the average number of substitutions per codon along all branches. dN/dS ratio is given for the codons under selection, along with the % of codons in this category.

The sequences were used to construct neighbor-joining trees (based on Kimura 2-parameter distances) for the near full-length sequences (Figure S1A) and for the 2–4 exon set (Figure 4A). Modifications to the trees were made based on generally accepted phylogenetic trees [29], [30]. The data-based and taxonomy-based trees were both used for PAML analysis and produced nearly identical statistics (Tables S2,S3). Values of dN/dS along each tree branch were calculated using the free-ratio model of PAML. A dN/dS value >1 suggests that positive selection has acted along that lineage. Several branches of the trees show evidence of positive selection with dN/dS>1, or, when dS = 0, by the identification of 4 or more replacement substitutions.

Likelihood ratio tests indicate that mA3 has a significant probability of having experienced positive selection, and this was the case for all codon frequency models, and for both datasets (Figures 4B and S1B, Tables S2, S3). The Bayes empirical Bayes calculation of posterior probabilities in PAML identified specific mA3 codon positions as having significant probability of positive selection. In the separate analyses of the two datasets, we identified 20 codons as being under positive selection with high posterior probability P>0.95, and 10 of these 20 codons were under very strong positive selection with P>0.99 (Tables 1, S2, S3). Sixteen of these 20 codons are in exons 2–4. Analysis of the smaller set of 8 near full-length genes identified a subset of the positively selected codons identified by analysis of exons 2–4. The full-length sequence analysis also identified 5 additional codons under positive selection with P>0.95 that were not identified in the exon 2–4 analysis: one codon, 142, in exon 3 of the active CDA and four codons, 201, 273, 316 and 371, in the inactive C-terminal CDA (Tables 1, S3).

**Table 1:** Sites that distinguish the mA3 genes in virus-restrictive C57BL and virus-nonrestrictive BALB/c mice, and sites under positive selection.

Codon	Residue		Posterior Probability of Positive Selection^b^	
	C57BL	BALB/c^a^	Exons 2–4 (25 mice)	Full length (8 mice)
25	T		**0.967**	0.453
34	G	R	**0.998**	**0.954**
37	K	I	**1.000**	**0.968**
38	G	D	**1.000**	**0.999**
60	H		**0.991**	0.684
112	I	V	0.105	0.468
113	V	L	**0.966**	**0.974**
128	S		0.946	**0.950**
134	V	I	**0.999**	0.781
135	Q	R	**1.000**	**0.999**
136	D		**0.999**	0.189
138	E		**0.976**	**0.974**
139	T	N	**0.980**	**0.952**
142	N		0.589	**0.974**
175	R		**0.976**	0.880
181	R	K	0.946	0.778
183	L		**0.977**	**0.982**
200	M	V	-	0.674
201	D	H	-	**0.993**
202	P	L	-	0.472
225	R	G	-	0.691
273	T	I	-	**0.979**
316	C		-	**0.971**
371	R	H	-	**0.993**

a The BALB/c residue is given where it differs from C57BL.

b Calculated using PAML for two sets of sequences. The probabilities were determined using Codon Table model F61 and selection Model 8 on the taxonomy-based trees. Sites under selection with probabilities >0.95 are in bold, and strongly selected sites with P>0.99 are also underlined. Use of the codon frequency model F3X4 identified selection with P>0.95 at codon 181 and with P>0.99 at codon 183 (Table S2).

**Figure 5:**
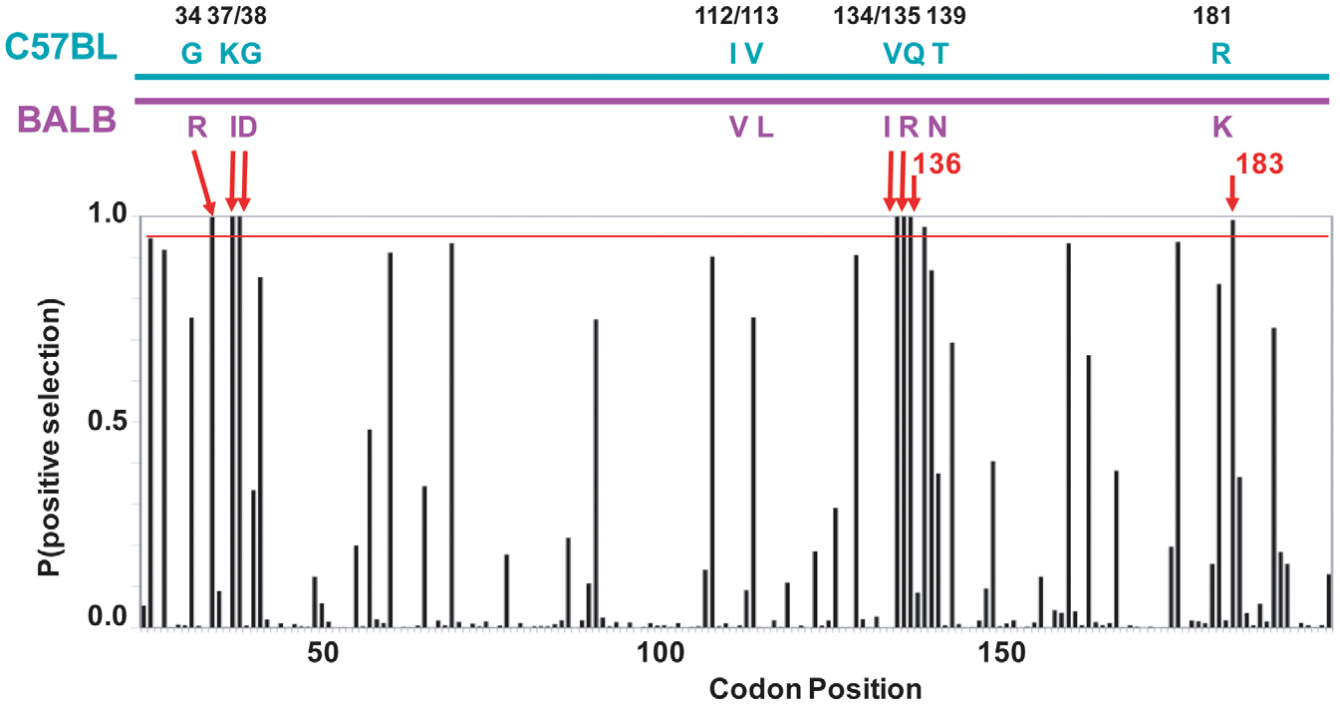
mA3 sites under positive selection in the N-terminal Z2 CDA. At the top is a diagram of the mA3 segment (exons 2–4) that encodes the N-terminal CDA showing the locations of the 9 codons that differ in virus restrictive C57BL and virus nonrestrictive BALB/c mice. At the bottom is a graph showing the posterior probability of positive selection at each codon based on an analysis of 26 mA3 sequences using codon frequency model F3X4 and selection Model 8 on the data-based trees (Table S2). A red line marks P = 0.95. The red arrows indicate the 7 codons that are strongly positively selected (P>0.99); these include 5 of the 9 polymorphic codons (long arrows) and codons 136 and 183. Codon 138 is positively selected at 0.99>P>0.95.

There are 15 mA3 codons that specify different amino acids in virus restrictive C57BL and sensitive BALB/c mice. Eleven of these codons were found to be under positive selection (P>0.95), and 5 of the codons under very strong positive selection (P>0.99) mapped to two clusters in the active CDA (Figure 5). Because this type of analysis is designed to identify sites involved in diversifying selection (antagonistic interactions with pathogens being a prime example), our results indicate that most of the residues that distinguish C57BL and BALB/c mice identify key sites likely to be involved in genetic conflicts. These results also suggest that mA3 has had a defensive role that predates development of the laboratory strains and involves species in all 4 *Mus* subgenera.

### Homology modeling

Homology models for the C57BL N-terminal active CDA sequences were chosen from the LOMETS homology modeling program based on templates that had the highest sequence identity. The search identified several templates with highest confidence, crystal structures determined for the catalytic domain of hA3G (PDB ID 3IR2) [31] and (PDB ID 3IQS, 3E1U) [32]. The hA3G-3IR2 template model, based on the active hA3G C-terminal Z1 deaminase domain, was chosen for detailed analysis because it provides more coverage of the N-terminal Z2 domain of the mouse sequence [23], [31], and because it was the top LOMETS solution overall.

**Figure 6:**
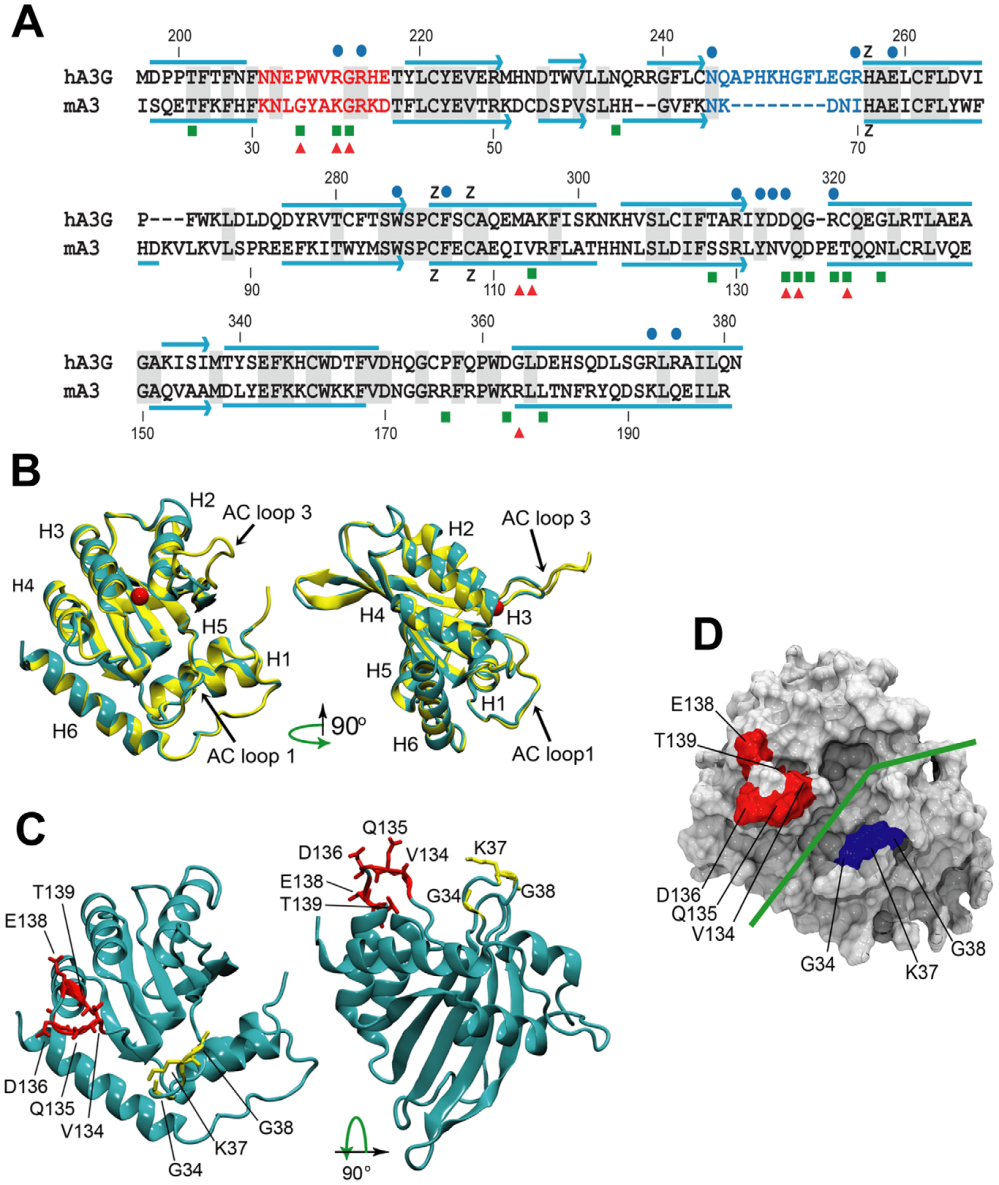
Comparative sequence and structure of the active cytidine deaminase regions of hA3G and mA3. A) Sequence alignment of the C57BL mA3 CDA (GenBank No. NM_030255) with that of hA3G (GenBank No. NM_021822), using CLUSTAL X [62]. Gray shading, identical residues. Red letters, AC LOOP 1. Blue letters, AC LOOP 3. Blue lines, α-helices. Blue arrows, β-strands. Blue dots, residues associated with the hA3G substrate groove that are important for deamination [32]–[34]. Green boxes, sites under positive selection in mA3. Red triangles, sites that are polymorphic between BALB/c and C57BL. B) Superimposed structures of hA3G-3IR2 and the mA3 homology model. α-helices are numbered. AC loops 1 and 3 are indicated. The red ball is zinc. C) Homology model of mA3 showing the 3 AC loop 1 residues under positive selection (yellow), and the 5 residues under strong (P>0.95) and very strong (P>0.99) selection that are on the opposite side of the substrate groove (red). D) Surface representation of mA3 showing the location of the predicted substrate groove (green line) and the two clusters of selected residues in blue and red.

The C57BL mA3 CDA sequence has 36.4% identity to the hA3G CDA (Figure 6A). Superposition of the hA3G-3IR2 crystal structure and the mouse homology model show they share the 5 stranded β-sheet core surrounded by 6 α-helices that is common to known deaminase structures, along with a conservation of active-site loops involved in substrate binding (Figure 6B). The sidechain conformations of the C57BL residues involved in coordinating Zn are identical to their counterparts in the hA3G structure (Figure 6A,6B). The overall fold between the human and mouse structures is nearly the same with the RMSD (root mean square deviation) between backbone atoms of the C57BL mA3 model and the human structure being 0.56Å. The RMSD between all atoms for the mouse model and the human structure is 0.94Å.

Mutagenesis, NMR DNA titration data and structural analysis of hA3G-3E1U and the NMR structure hA3G-2JYW have identified key residues important in deaminase activity and formation of the substrate groove [32]–[34]. Among these key hA3G sites are the catalytic E259, 3 hydrophobic residues and 10 critical residues of which 9 are charged, all of which are within and brimming the groove and all of which are needed for deaminase activity (Figure 6A). N244 and R256 are associated with active center loop 3 (AC loop 3), R213 and R215 are present in active center loop 1 (AC loop 1), residue R313 resides on the floor of the groove and D316, D317, R320 face the substrate groove at or near the end of helix 4. The most obvious difference between hA3G-3IR2 and the mouse model in these functionally important sites is the presence of an 8 residue deletion in the AC loop 3 of the mouse model. hA3G AC loop 3 is an unstructured loop, and the deletion of the majority of the residues in the mouse AC loop 3 suggests they play no critical role; the mouse AC loop 3 structure, however, does conserve the two residues found at the hA3G loop base, N244 and R256, known to be critical for deamination [32], and it is likely that these mouse residues, N66 and I70, serve similar functions in mA3.

In contrast to this difference in AC loop 3, the functionally important AC loop 1 and helix 4 residues in hA3G are retained in mA3, and closely align with the two clusters of residues in mA3 shown here to be under positive selection (Figure 6A, 6B, 6C). On the other side of the substrate groove from selected AC loop 1 residues 34–38 is the region encompassing residues 134–139 in C57BL (and the corresponding region in hA3G); these residues are at the end of helix 4 with some residues participating in the α-helix and the rest as a loop. A solvent accessible surface representation of the mA3 structure indicates the position of the predicted substrate groove, and suggests the location of the two clusters of positively selected residues on opposite sides of this substrate groove (Figure 6D). The residues at the end of helix 4 and the residues in the 34–38 cluster on the other side of the mA3 groove likely serve steric roles in maintaining groove structure and likely also have functional roles based on charge and hydrophobicity that govern substrate interactions.

### *In vitro* antiviral activity of mA3 mutants

**Figure 7:**
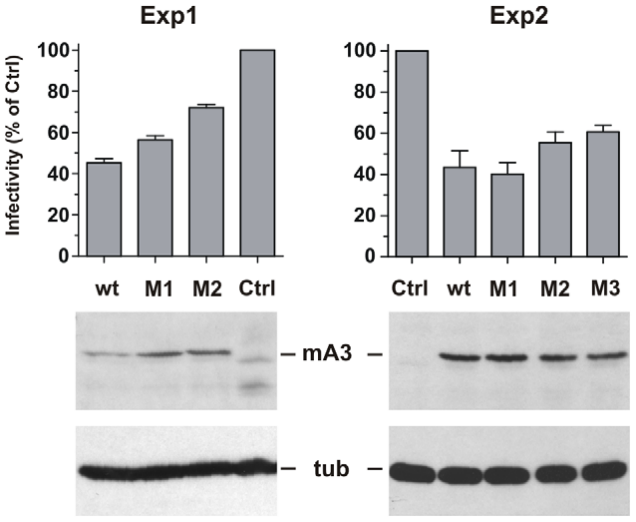
Antiviral activity of mA3 mutants carrying BALB/c residues at the 34–38 and 134–139 clusters. 293T cells were cotransfected with Friend virus clone pLRB302 and the indicated mA3 variants. The experiment was repeated six times with some experimental variations; shown are two representative experiments. One experiment (left panel) used 3 µg of pLRB302 and 1 µg of mA3. The second experiment (right panel) used 4 µg of pLRB302 and 0.5 µg of mA3, and total DNA amounts were adjusted to 6 µg with empty pcDNA3.1(−) vector DNA; mA3 was reduced because titration experiments showed 0.5 µg to have antiviral activity (not shown). Infectivity of collected Friend virus was assayed by the XC overlay test normalized against reverse transcriptase activity (experiment 1) or virus capsid protein in pelleted virus (experiment 2). Infectivity of virus produced in the absence of mA3 was defined as 100%, and infectivity of the other viruses is given as a percentage of the control. Virus infectivity was determined 6 times in both experiments.

293T cells were cotransfected with the pLRB302 Friend virus clone and mA3 clones to assess the relative antiviral activities of 4 mA3 clones: the wild type *Rfv3* virus resistant C57BL mA3 [13] and three clones with mutations that introduced residues of the *Rfv3* virus sensitive BALB/c: M1 (G34R, K37I, G38D), M2 (V134I, Q135R, T139N), M3 (all 6 substitutions) (Figure 7). Cells and virus-containing supernatants were harvested 48 hours after transfection. Cells were analyzed by immunoblotting for mA3 expression, and infectious virus in the supernatants was quantitated by the XC overlay test. For each of the transfected mA3 clones, infectious virus titers decreased in a dose dependent manner relative to increasing expression of mA3 (data not shown). The wild type C57BL mA3 and the BALB-like M3 mutant both showed antiviral activity, but the antiviral activity of M3 was reduced relative to wild type mA3 (Figure 7). The M1 mutant mA3 was found to reduce the infectivity of Friend virus as effectively as wild type C57BL mA3, whereas M2 more closely resembled M3 in antiviral activity suggesting that substitutions in the 134–139 cluster are particularly important for anti-FrMLV activity.

## Discussion

This analysis indicates that mA3 has been involved in genetic conflicts through *Mus* evolution. This gene shows strong positive selection marked by an increase in replacement versus synonymous substitutions. Six of the 10 codons that evolved under strongest positive selection are in two clusters in the N-terminal catalytically active CDA. Five of these 6 codons specify different amino acids in MLV and MMTV restrictive and nonrestrictive mouse strains, and mutational analysis suggests these residues contribute to antiviral activity. We also demonstrate that the antiviral allelic variant has acquired a retroviral LTR insertion, the presence of which is associated with elevated mA3 expression levels in the spleens of inbred and wild-derived mice.

Retroviral insertions can be important functional components of the host genome, and can clearly affect host gene expression. Examination of spontaneous mutations in the mouse suggested that 10–12% of all mutations are due to ERV insertions [35]. Like the mA3 LTR, most of these mutant-associated ERVs are in reverse orientation in introns, and the responsible mutational mechanisms include two of relevance here: aberrant splicing and enhanced transcription driven by the ERV LTR. While the mA3 LTR is inserted at a splice donor site, it does not alter splicing of the associated intron, and although all mice carrying this LTR produce the same Δexon5 mA3 isoform, the absence of this LTR in at least one mouse species producing that isoform (*M. m. molossinus*) suggests that the LTR was acquired by mice already preferentially producing this splice variant. As for LTR-driven altered expression levels, two of three previous studies that compared mA3 RNA levels in virus-resistant and susceptible strains reported that mA3 expression levels are significantly higher in mice carrying the LTR+ C57BL allele compared to LTR− BALB/c [21], [17], [10]. Our analysis of mA3 expression levels shows a correlation between the presence of the LTR and elevated expression in a variety of inbred strains and mouse species. Because enhancer activation of cellular genes by viral LTRs can occur with insertions in either orientation and at considerable distance from the cellular promoter, it is thus possible that the enhancer of this inserted LTR sequence drives the elevated expression observed in the LTR+ mice. This elevated expression in conjunction with altered splicing may together have contributed to the evolution of the antiviral C57BL mA3. It has been suggested that the Δexon5 isoform has enhanced antiviral activity due to its resistance to the viral protease [36]; elevated expression of this variant due to subsequent LTR insertion would further boost the survival value of this factor.

It is particularly intriguing that this X-MLV LTR sequence is found in NZB and CZECH mice and one breeding line of *M. m. castaneus*. These mice are unusual among laboratory strains and wild mice in that they harbor highly active X-MLV ERVs producing infectious virus, and such active ERV expression increases the likelihood of insertional mutagenesis. NZB mice are characterized by lifelong viremia with X-MLVs [37]. *M. m. castaneus* and CZECH mice are among wild mouse Eurasian populations with highest copy number of X-MLV ERVs [38], and we have isolated infectious X-MLV-related virus from both of these wild mice [39], [40]. If in fact the inserted MLV LTR causes elevated mA3 expression, then this would provide another instance of an ERV sequence that is co-opted by the virus-infected host for an antiviral function, other examples in the mouse being *Fv1*, *Fv4*, and *Rmcf* [41].

In addition to differences in splicing and expression levels, mA3 genes of virus resistant and sensitive mice differ in protein sequence. Our phylogenetic analysis showed that most of these polymorphic sites are under strong positive selection. The alignment of these sites with functionally important residues in the hA3G C-terminal active CDA suggests they serve similar roles in the mouse and that therefore, this function has been important during *Mus* evolution. That this evolutionarily important function is related to mA3 deaminase activity is supported by the observation that the great majority of these selected residues are in the N-terminal half of mA3 which encodes the active Z2 CDA [22] and that antiviral activity resides in the first 194 amino acids (exons 1–4) [10]. In the predicted mA3 structure, these positively selected residues are positioned in one of two loops assigned functional importance in hA3G, AC loop 1 and a cluster of residues facing AC loop 1 on the other side of the putative substrate groove [32]–[34]. The charged and hydrophobic residues in these regions are positioned to maintain structural integrity of the groove and to interact with one another and the nucleic acid substrate in a way that could contribute to substrate specificity.

Three positively selected residues, G34, K37 and G38, in the mA3 AC loop 1 sequence KNLGYAKGRKD are most likely responsible for providing conformational freedom (in the case of the G34 and G38) and for interacting favorably with the phosphate backbone (in the case of K37). The electrostatic contributions of K37 along with K40 and D41 probably play an important role in determining substrate affinity and specificity while Y35 is in a position to stack with a nucleotide base. The analogous sequence in hA3G is NNEPWVRGRHE (207–217) with R213, H216 and E217 positioned to interact electrostatically with a phosphate backbone and W211 able to stack with a nucleotide base. R39 (mA3) and R215 (hA3G) are positioned similarly in that the residue provides an elaborate H-bonding network defining the shape of AC loop 1 [32].

Five positively selected residues (V134, Q135, D136, E138 and T139) lie in a region that comprises the end of helix 4 and an adjacent loop that define the side of the substrate binding groove opposite of AC loop 1 (mA3 sequence YNVQDPET). Close inspection of this region in the mouse model reveals that the sidechain of D136 is in a position to H-bond with T139 maintaining the helical nature of helix 4 despite the presence of P137. This has the result of allowing Q135 to form the top-side of the groove allowing V134, N133 and Y132 to form the side of the groove with Y132 in position to stack with a nucleotide base. Y132 is invariant in our mouse sequences along with nearby W102 which defines the floor of the groove. The homologous segment of human APOBECs has now been implicated in the distinctive substrate preferences among AID/APOBEC family members which target cytosine within different sequence motifs. A recognition loop responsible for these preferences (hA3G sequence IYDDQGRCQ) lies between the β4 strand and the α4 helix (Figure 6A, residues 314–322) [42]. That this highly variable region controls substrate preferences is also supported by mutational analysis [32], [43]. Alignment of the active CDAs of hA3G and mA3 indicates that this loop overlaps the 134–139 cluster of positively selected residues in mA3. This suggests that genetic conflicts between host and pathogen in this case produced positive selection that may be driven, not by protein-protein interactions, but by the interaction of mA3 and varying ssDNA substrates, a suggestion that is also consistent with the finding that the efficiency of substrate deamination is sensitive to ssDNA secondary structure [44].

Mutational analysis of 6 codons in the two clusters under positive selection showed that introduction of BALB/c residues, particularly in the 134–139 cluster, reduced antiviral activity against Friend MLV. Further studies may determine if the differences associated with overexpressed mA3 in transiently transfected cells have physiological relevance, and whether substitutions at these sites similarly affect restriction of other retroviruses. It has been reported that mA3 shows stronger antiviral activity against HIV-1 than against MLV [45], suggesting that the genetic conflicts responsible for positive selection during *Mus* evolution may have resulted from interactions with pathogens unrelated to the FrMLV used here.

Previous phylogenetic analysis of hA3G had identified 21 sites under very strong positive selection, 9 of which are in the active CDA [46]. One of these sites, R213, aligns with one of the clusters of residues (positions 34–38) under strong selection in mA3; however, the analysis of hA3G did not identify selection in the region aligning with the second cluster under strong selection in mA3 (positions 134–139), although this segment is a substrate recognition loop that is highly variable among members of the AID/APOBEC family [42]. The additional sites identified to be under positive selection in the hA3G active CDA have no positively selected counterparts in mA3. Among these additional sites in hA3G, two, H248 and K249, lie in AC loop 3 [46]. Mutagenesis and analysis of hA3G structure have implicated this loop in antiviral deamination [32], but much of AC loop 3 is deleted in the mouse, leaving only the key residues at the base of this loop that align with critical residues N244 and R256. The residues at these sites are invariant in our mA3 sequences suggesting their evolution is under purifying selection. The differences in AC loop 3 between hA3G and mA3 and the fact that different residues are under selection in hA3G and mA3 suggests there may be functional differences between these proteins.

Our analysis of the full-length mA3 sequences also identified four sites under positive selection in the C-terminal half of the protein (Tables 1, S1) that carries the Z3 CDA that has been determined to be inactive [22]. It is not clear what role these residues serve. An antiviral role for the C-terminal half of mA3 is suggested by the observation that that the conserved glutamates in the N-terminal Z2 domain and the C-terminal Z3 domain of mA3 are both required for antiviral activity against HIV-1 [45]. Other evidence suggests that the inactive CDA is involved in virus encapsidation [47]. We note that alignment of the mouse Z2 and Z3 CDA regions shows that one of the two selected Z3 codons, P316, aligns with the 134–139 selected cluster of codons in Z2, VQDPET. Another selected codon in the Z3 CDA, T273, aligns with an hA3G segment with two codons under selection in primates [46]. This suggests the possibility that this Z3 CDA may have had deaminase activity in some branches of the *Mus* lineage.

Further analysis of the C57BL and BALB/c mA3 genes should shed light on the functional roles of the polymorphic residues in the two groove-associated clusters. The information from additional phylogenetic, structural, and functional comparisons will help describe the range of antiviral activity and evolutionary history of this gene. We are currently analyzing additional mA3 mutants for antiviral activity, and using molecular dynamics simulations to describe the structural implications of specific substitutions.

## Methods

### Genomic DNA and RNA

DNA and RNA were isolated from animals and cell lines developed from laboratory mouse strains and from wild mice and wild mouse-derived breeding colonies (Table S1). Many wild-derived mice were obtained from M. Potter (NCI, Bethesda, MD). SAMP8 mice were provided by R. Carp (New York State Institute for Basic Research in Developmental Disabilities, Staten Island, NY), SIM.S mice were obtained from E. Boyce (Memorial Sloan-Kettering Cancer Center, NY), and mice trapped in California were provided by S. Rasheed (University of Southern California, Los Angeles). Mice or DNA samples of *M. spretus* (SPRET/EiJ), *M. m. castaneus* (CAST/EiJ), various inbred lines derived from *M. m. molossinus*, PERA, PERC, PWD, and the inbred strains listed in Figure 2 were obtained from The Jackson Laboratory (Bar Harbor, ME).

A set of African pygmy mouse DNA samples was obtained from Y. Cole and P. D'Eustachio (Depts. Biochemistry and Medicine, NYU, New York); these mice had been classed into 4 species of subgenus *Nannomys* mice on the basis of skeletal features by J. T. Marshall (Smithsonian Natural History Museum, Washington, DC). A sample of *M. m. macedonicus* DNA was provided by R. Elliott (Roswell Park, Buffalo). Cell lines used as DNA and RNA sources included NZB-Q and *M. fragilicauda* cells obtained from J. Hartley (NIAID, Bethesda, MD), cells from some wild mouse species obtained from J. Rodgers (Baylor College of Medicine, Houston, TX), and NIH 3T3, *M. dunni* [48], SC-1 [49], A9 (C3H/He) [50], and CMT93 (C57BL) (ATCC CCL-223).

### Ethics statement

All studies in which animals are involved were performed in accordance with the guidelines of the Committee on the Care and Use of Laboratory Animals under an NIAID-approved animal study protocol [51], and all studies and procedures were reviewed and approved by the Institutional Animal Care and Use Committee of the NIH.

### *APOBEC3* sequences of wild mouse species

*APOBEC3* segments were amplified from mouse genomic DNAs or RNAs using primers designed from coding, flanking or intron sequences based on the C57BL genomic sequence (GenBank No. NT_03921) (Figure 1A). Exon 2 was amplified using forward intron primer a: 5′-CTCCTCTCCCTCTGTCTTCCT and reverse primer b: 5′-GGATTCAAGGTATGAGCCACCATGC. Exons 3 and 4 were amplified using primer c: 5′-GCTTCAACAGGGCTCAGAGTGC and primer d: 5′-AGGTTTGGGAGGAGGGAGAAC. Reverse transcription PCR (RT-PCR) was used to amplify near full-length APOBEC3 from total RNA using primer e in exon 1 (5′-GGACCATTCTGTCTGGGATGCAGCCATCG) and primer f in exon 9 (5′-GACATCGGGGGACCAAGCTGTAGGTTTCC) and a shorter RT-PCR fragment was generated using primer a and primer g (5′-GGTTGTAAAACTGCGAGTAAAATTCC). The larger RT-PCR product contained 1083 bp of the full-length 1287 bp mA3 sequence. Most of these products lacked the 99 bp exon 5, and the aligned sequences lack 72 bp at the 5′ end and 33 bp at the 3′ end of the gene. PCR products were sequenced directly in some cases, and in others fragments were first cloned into pCR2.1-TOPO (Invitrogen, Carlsbad, CA) before sequencing (Text S1, S2).

### Real-time PCR

Total RNAs from mouse spleens were isolated using Trizol (Invitrogen). Reverse transcription was carried out at 50°C for 1 hour using 2 µg of total RNA in the presence of Oligo (dT) primer (Ambion, Austin, TX) and SuperScript III (Invitrogen). After reverse transcription, the reaction mixtures were diluted to 1000 µl with DEPC-water. 1 µl of the diluted cDNA were added to a 15 µl PCR reaction mix containing 0.4 µl of 10 µM primers and 2× SYBR Green PCR mix (Applied Biosystems, Foster City, CA ). APOBEC3 transcripts were amplified using primers 5′-GACCATTCTGTCTGGGATGCA and 5′-TTCTAGTCACTTCATAGCACA. β-actin was also measured using primers (5′- GTGGGGCGCCCCAGGCACCA; 5′- CTCCTTAATGTCACGCACGATTTC) as a normalization control. Amplification was done under the condition of 15 s at 95°C and 1 min at 60°C for 50 cycles in a 7300 Real Time PCR System (Applied Biosystems).

### Antiviral activity of mA3 sequence variants *in vitro*

HA-tagged mA3 [13] was obtained from the NIH AIDS Research and Reference Reagent Program (Germantown, MD) (catalog no. 10021) and mutagenized using the QuikChange mutagenesis kit (Stratagene, La Jolla, CA) to introduce substitutions at 6 codons. M1 (G34R, K37I, G38D) was generated using primer 5′-CCACTTTAAGAACCTACGCTATGCCATTGATCGGAAAGATACCTTC and its reverse complement. M2 (V134I, Q135R, T139N) was generated using primer 5′-GCTCCCGCCTCTACAACATCCGAGACCCAGAAAATCAGCAGAATCTTTGC and its reverse complement. M3, containing mutations at all 6 codons, was generated by mutating M1 with the primers designed for M2. Mutations were confirmed by sequencing. Attempts to generate stable transfectants of various mouse cells expressing these mA3 variants were not successful. Human 293T cells were co-transfected with 3–4 µg of the pLRB302 clone of Friend MLV [52] obtained from L. Evans (RML, NIAID, Hamilton, MT), and 0.5 or 1.0 µg mA3. At 48 hours after transfection, the culture supernatant was collected and virus infectivity was measured by the XC overlay test [53]. In this test, subconfluent cultures of NIH 3T3 cells were infected with virus dilutions, irradiated 4 days later and overlaid with rat XC cells. Infectivity was determined as plaque-forming units per ml of culture fluid. Infectivity was normalized against reverse transcriptase activity [54] or virus-associated capsid protein in pelleted virus. After electrophoresis on 12.5% SDS-polyacrylamide genes and transfer to polyvinylidene difluoride membranes, capsid protein was detected using polyclonal goat anti-Rauscher MLV p30 antiserum (Viromed Biosafety Laboratories (NCI/BCB Repository), Camden, NJ) and horseradish peroxidase conjugated rabbit anti-goat antibody (Invitrogen catalog # R21459). The transfected 293T cells were lysed and tested for mA3 expression by western immunoblot analysis. Cell lysates were subjected to electrophoresis and western blots were probed with a monoclonal antibody against HA, HA-7 (Sigma catalog #H-3663) and a monoclonal anti-tubulin antibody (Sigma #T-9026).

### Selection analysis of lineages and codons

DNA sequences were aligned using MUSCLE [55] and improved manually. Two phylogenies were produced, one for the full-length sequences and one for the exon 2–4 sequences. In all cases the Kimura 2-parameter distance-based neighbor-joining phylogenies for each set returned by PHYLIP (version 3.68) [56]) were corrected for closer correspondence to the consensus *Mus* phylogeny [29], [30]. The trees were corrected to make the *Nannomys* species a monophyletic group and to place *M. spretus* basal to the *M. musculus* node.

The codeml program of the PAML4 package [28] was used for maximum likelihood analysis of codon evolution [57]. Both lineage-specific and codon-specific analyses were performed. In the lineage-specific selection analyses, the free ratio model (codon model = 1) was used to calculate branch-specific rates of dN/dS. In this model each branch is assumed to have a specific dN/dS ratio. The likelihood of the phylogeny under this model was tested against the likelihood of the phylogeny under the model of one uniform dN/dS ratio across all branches (codon model 0) using a likelihood ratio test (LRT). The significance of the LRT value was assessed using a chi-squared distribution with 49 degrees of freedom for the exon 2–4 sequence analysis and 12 degrees of freedom for the full-length sequence analysis.

Selection acting on *Apobec3* codons was analyzed using two models of equilibrium codon frequencies and four models of codon selection. The two codon frequency models used were the F3x4 model (codon frequencies estimated from the nucleotide frequencies in the data at each codon site) and the F61 (Codon Table) model (frequencies of each of the 61 non-stop codons estimated from the data). The codon selection models were two neutral/negative selection models (M1 and M7) which were compared against corresponding positive selection models which included a category for dN/dS>1 (M2 and M8, respectively). The significance of this additional codon selection category was assessed using LRTs of the phylogeny likelihoods under the neutral and positive selection models. Significance of the test statistics was calculated using a chi-squared distribution with two degrees of freedom. The Bayes empirical Bayes algorithm [58] was used to calculate the posterior probability of individual codons experiencing dN/dS>1.

### Homology modeling

The C57BL mouse mA3 sequence (GenBank No. NM_030255) was submitted to the LOMETS program [59]. A model constructed using a template with the highest sequence identity was chosen from the top ten solutions ranked by a combination of highest sequence identity, most coverage, Z-score and overall confidence. The model was generated using Modeller v4 [60] and energy optimized in SYBYL7.3 using the AMBER7 ff99 forcefield with AMBER7 ff99 atom types and charges with the Powell method to a termination gradient of 0.05 kcal/mol·Å. The model was examined using Procheck [61] to detect any bad geometries.

### Accession numbers

mA3 exon 2–4 sequences were given GenBank Accession Nos. GQ901957–GQ901974. Near full length sequences were given GenBank Nos. GQ871500–506.

## Supporting Information

Table S1 Designations and sources of wild-derived mice, cells and DNAs.
(0.08 MB DOC)

Table S2 PAML summary results for mA3 exons 2–4.
(0.10 MB DOC)

Table S3 PAML summary results for *Apobec3* full-length sequence.
(0.10 MB DOC)

Figure S1 Phylogenetic tree and likelihood ratio tests for the mA3 full length sequence. A) Data-derived cladogram showing branch values of dN/dS calculated using the free-ratio model of PAML, with the number of replacement and synonymous changes in parentheses. When dS = 0, dN/dS is infinite (Inf). dN/dS>1 suggests positive selection along that lineage. B) Likelihood ratio tests were used to tests for positive selection. Neutral models (M1, M7) were compared with selection models (M2, M8) using two different models of codon frequency (F3X4 or F61). P values <0.0001 provide strong evidence of positive selection. Tree 1 is the data-derived tree and tree 2 is the taxonomy-derived tree. Tree length is the average number of substitutions per codon along all branches. dN/dS ratio is given for the codons under selection, along with the % of codons in this category.
(0.47 MB TIF)

Text S1 Alignment of full-length mA3 sequences from mice listed in Table S1. The codons under positive selection by PAML are boxed, with red boxes indicating codons under very strong selection (P>.99). Green fill marks codons that distinguish C57BL and BALB/c. Selection analysis by maximum likelihood is limited to sites typed in all DNAs and excludes 72 bp at the 5′ end, 33 bp at the 3′ end and the 99 bp exon 5. The C57BL mA3 sequence was from GenBank (No. NM_030255). The sequence derived from NIH 3T3 was used in place of BALB/c as all clones from BALB 3T3 cells lacked exons 2 and 5, and the NIH 3T3 sequence was otherwise identical to the published BALB/c sequence (GenBank No. EDL04624).
(0.07 MB DOC)

Text S2 Alignment of mA3 Exon 2–4 sequences from mice listed in Table S1. The codons under positive selection by PAML are boxed, with red boxes indicating codons under very strong selection (P>.99). Green fill marks codons that distinguish C57BL and BALB/c. The C57BL mA3 sequence was from GenBank (No. NM_030255). BALB/c exons 3 and 4 were amplified from mRNA and exon 2 from DNA.
(0.08 MB DOC)

## References

[1] Lilly F (1967). Susceptibility to two strains of Friend leukemia virus in mice.. Science.

[2] Best S, LeTissier P, Towers G, Stoye JP (1996). Positional cloning of the mouse retrovirus restriction gene *Fv1*.. Nature.

[3] Yan Y, Buckler-White A, Wollenberg K, Kozak CA (2009). Origin, antiviral function and evidence for positive selection of the gammaretrovirus restriction gene *Fv1* in the genus *Mus*.. Proc Natl Acad Sci U S A.

[4] Keckesova Z, Ylinen LM, Towers GJ (2004). The human and African green monkey Trim5α genes encode Ref1 and Lv1 retroviral restriction factor activities.. Proc Natl Acad Sci U S A.

[5] Stremlau M, Owens CM, Perron MJ, Kiessling M, Autissier P (2004). The cytoplasmic body component Trim5α restricts HIV-1 infection in Old World monkeys.. Nature.

[6] Tareen SU, Sawyer SL, Malik HS, Emerman M (2008). An expanded clade of rodent *Trim5* genes.. Virology.

[7] Goila-Gaur R, Strebel K (2008). HIV-1 Vif, APOBEC, and intrinsic immunity.. Retrovirology.

[8] Harris RS, Bishop KN, Sheehy AM, Craig HM, Petersen-Mahrt SK (2003). DNA deamination mediates innate immunity to retroviral infection.. Cell.

[9] Newman ENC, Holmes RK, Craig HM, Klein KC, Lingappa JR (2005). Antiviral function of APOBEC3G can be dissociated from cytidine deaminase activity.. Curr Biol.

[10] Takeda E, Tsuji-Kawahara S, Sakamoto M, Langlois M-A, Neuberger MS (2008). Mouse APOBEC3 restricts Friend leukemia virus infection and pathogenesis in vivo.. J Virol.

[11] Kao S, Khan MA, Miyagi E, Plishka R, Buckler-White A (2003). The human immunodeficiency virus type 1 Vif protein reduces intracellular expression and inhibits packaging of APOBEC3G (CEM15), a cellular inhibitor of virus infectivity.. J Virol.

[12] Esnault C, Heidmann O, Delebecque F, Dewannieux M, Ribet D (2005). APOBEC3G cytidine deaminase inhibits retrotransposition of endogenous retroviruses.. Nature.

[13] Mariani R, Chen D, Schrofelbauer B, Navarro F, Konig R (2003). Species-specific exclusion of APOBEC3G from HIV-1 virions by Vif.. Cell.

[14] Okeoma CM, Lovsin N, Peterlin BM, Ross SR (2007). APOBEC3 inhibits mouse mammary tumour virus replication *in vivo*.. Nature.

[15] Mikl MC, Watt IN, Lu M, Reik W, Davies SL (2005). Mice deficient in APOBEC2 and APOBEC3.. Mol Cell Biol.

[16] Jern P, Stoye JP, Coffin JM (2007). Role of APOBEC3 in genetic diversity among endogenous murine leukemia viruses.. PLoS Genet.

[17] Santiago ML, Montano M, Benitez R, Messer RJ, Yonemoto W (2008). *Apobec3* encodes *Rfv3*, a gene influencing neutralizing antibody control of retrovirus infection.. Science.

[18] Chesebro B, Wehrly K (1979). Identification of a non-*H-2* gene (*Rfv-3*) influencing recovery from viremia and leukemia induced by Friend virus complex.. Proc Natl Acad Sci U S A.

[19] Doig D, Chesebro B (1979). Anti-Friend virus antibody is associated with recovery from viremia and loss of viral leukemia cell-surface antigens in leukemic mice. Identification of Rfv-3 as a gene locus influencing antibody production.. J Exp Med.

[20] Super HJ, Hasenkrug KJ, Simmons S, Brooks DM, Konzek R (1999). Fine mapping of the Friend retrovirus resistance gene, *Rfv3*, on mouse chromosome 15.. J Virol.

[21] Okeoma CM, Petersen J, Ross SR (2009). Expression of murine APOBEC3 alleles in different mouse strains and their effect on mouse mammary tumor virus infection.. J Virol.

[22] Hakata Y, Landau NR (2006). Reversed functional organization of mouse and human APOBEC3 cytidine deaminase domains.. J Biol Chem.

[23] LaRue RS, Andresdottir V, Blanchard Y, Conticello SG, Derse D (2009). Guidelines for naming nonprimate APOBEC3 genes and proteins.. J Virol.

[24] Elder JH, Gautsch JW, Jensen FC, Lerner RA, Chused TM (1980). Differential expression of two distinct xenotropic viruses in NZB mice.. Clin Immunol Immunopath.

[25] O'Neill RR, Buckler CE, Theodore TS, Martin MA, Repaske R (1985). Envelope and long terminal repeat sequences of a cloned infectious NZB xenotropic murine leukemia virus.. J Virol.

[26] Beck JA, Lloyd S, Hafezparast M, Lennon-Pierce M, Eppig JT (2000). Geneologies of mouse inbred strains.. Nat Genet.

[27] Yang H, Bell TA, Churchill GA, de Villena FP-M (2007). On the subspecific origin of the laboratory mouse.. Nature Genet.

[28] Yang Z (1997). PAML: a program package for phylogenetic analysis by maximum likelihood.. Comput Appl Biosci.

[29] Lundrigan BL, Jansa SA, Tucker PK (2002). Phylogenetic relationships in the genus *Mus*, based on paternally, maternally, and biparentally inherited characters.. Syst Biol.

[30] Veyrunes F, Dobigny G, Yang F, O'Brien PCM, Catalan J (2006). Phylogenomics of the genus *Mus* (Rodentia: Muridae); extensive genome repatterning is not restricted to the house mouse.. Proc R Soc B.

[31] Shandilya SMD, Nalam MNL, Nalivaika EA, Gross PJ, Valesano JC (2010). Crystal structure of the APOBEC3G catalytic domain reveals potential oligomerization interfaces.. Structure.

[32] Holden LG, Prochnow C, Chang YP, Bransteitter R, Chelico L (2008). Crystal structure of the anti-viral APOBEC3G catalytic domain and functional implications.. Nature.

[33] Chen K-M, Harjes E, Gross PJ, Fahmy A, Lu Y (2008). Structure of the DNA deaminase domain of the HIV-1 restriction factor APOBEC3G.. Nature.

[34] Chen K-M, Martemyanova N, Lu Y, Shindo K, Matsuo H (2007). Extensive mutagenesis experiments corroborate a structural model for the DNA deaminase domain of APOBEC3G.. FEBS Lett.

[35] Maksakova IA, Romanish MT, Gagnier L, Dunn CA, van de Lagemaat LN (2006). Retroviral elements and their hosts: insertional mutagenesis in the mouse germ line.. PLoS Genet.

[36] Abudu A, Takaori-Kondo A, Isumi T, Shirakawa K, Kobayashi M (2006). Murine retrovirus escapes from murine APOBEC3 via two distinct novel mechanisms.. Curr Biol.

[37] East J, Tilly RJ, Tuffrey M, Harvey JJ (1978). The early appearance and subsequent distribution of murine leukaemia virus in NZB embryos.. Int J Cancer.

[38] Kozak CA, O'Neill RR (1987). Diverse wild mouse origins of xenotropic, mink cell focus-forming and two types of ecotropic proviral genes.. J Virol.

[39] Yan Y, Knoper RC, Kozak CA (2007). Wild mouse variants of envelope genes of xenotropic/polytropic mouse gammaretroviruses and their XPR1 receptors elucidate receptor determinants of virus entry.. J Virol.

[40] Yan Y, Liu Q, Kozak CA (2009). Six host range variants of the xenotropic/polytropic gammaretrovirsues define determinants for entry in the XPR1 receptor.. Retrovirology.

[41] Stocking C, Kozak CA (2008). Murine endogenous retroviruses.. Cell Mol Life Sci.

[42] Kohli RM, Abrams SR, Gajula KS, Maul RW, Gearhart PJ (2009). A portable hot spot recognition loop transfers sequence preferences from APOBEC family members to activation-induced cytidine deaminase.. J Biol Chem.

[43] Langlois MA, Beale RCL, Conticello SG, Neuberger MS (2005). Mutational comparison of the single-domained APOBEC3C and double-domained APOBEC3F/G anti-retroviral cytidine deaminases provides insight into their DNA target site specificities.. Nucleic Acids Res.

[44] Larijani M, Martin A (2007). Single-stranded DNA structure and positional context of the target cytidine determine the enzymatic efficiency of AID.. Mol Cell Biol.

[45] Jonsson SR, Hache G, Stenglein MD, Fahrenkrug SC, Andresdottir V (2006). Evolutionarily conserved and non-conserved retrovirus restriction activities of artiodactyl APOBEC3F proteins.. Nucleic Acids Res.

[46] Sawyer SL, Emerman M, Malik HS (2004). Ancient adaptive evolution of the primate antiviral DNA-editing enzyme APOBEC3G.. PLoS Biol.

[47] Browne EP, Littman DR (2008). Species-specific restriction of Apobec3-mediated hypermutation.. J Virol.

[48] Lander MR, Chattopadhyay SK (1984). A *Mus dunni* cell line that lacks sequences closely related to endogenous murine leukemia viruses and can be infected by ecotropic, amphotropic, xenotropic, and mink cell focus-forming viruses.. J Virol.

[49] Hartley JW, Rowe WP (1975). Clonal cell lines from a feral mouse embryo which lack host-range restrictions for murine leukemia viruses.. Virology.

[50] Clements GB, Fenyo EM, Klein G (1976). *In vitro* derived mouse A9 cell clones differing in malignancy: analysis by somatic cell hybridization with YACIR lymphoma cell clones.. Proc Natl Acad Sci U S A.

[51] NRC (1996). Guide for the care and use of laboratory animals.

[52] Portis JL, McAtee FJ, Kayman SC (1992). Infectivity of retroviral DNA in vivo.. J Acquired Immune Deficiency Syndrome.

[53] Rowe WP, Pugh WE, Hartley JW (1970). Plaque assay techniques for murine leukemia viruses.. Virology.

[54] Wu T, Lee CG, Buckler-White A, Kozak CA (2002). Genetic control of a mouse serum lipoprotein factor that inactivates murine leukemia viruses: evaluation of apolipoprotein F as a candidate.. J Virol.

[55] Edgar RC (2004). MUSCLE: multiple sequence alignment with high accuracy and high throughput.. Nucl Acids Res.

[56] Felsenstein J (2004). PHYLIP (Phylogenetic Inference Package) version 3.6.

[57] Bielawski JP, Yang Z, Nielsen R (2005). Maximum likelihood methods for detecting adaptive protein evolution.. Statistical Methods in Molecular Evolution.

[58] Yang Z, Wong WSW, Nielsen R (2005). Bayes empirical Bayes inference of amino acid sites under positive selection.. Mol Bio Evol.

[59] Wu S, Zhang Y (2007). LOMETS: A local meta-threading-server for protein structure prediction.. Nucleic Acids Research.

[60] Sali A, Blundell TL (1993). Comparative protein modelling by satisfaction of spatial restraints.. J Mol Biol.

[61] Morris AL, MacArthur MW, Hutchinson EG, Thornton JM (1992). Stereochemical quality of protein structure coordinates.. Proteins.

[62] Thompson JD, Gibson TJ, Plewniak F, Jeanmougin F, Higgins DG (1997). The CLUSTAL_W windows interface: flexible strategies for multiple sequence alignment aided by quality analysis tools.. Nuc Acids Res.

