# Supplementary material for: Adaptive Evolution of Mus Apobec3 Includes Retroviral Insertion and Positive Selection at Two Clusters of Residues Flanking the Substrate Groove
Source: PLoS Pathog. 2010 Jul 1;6(7):e1000974. doi: 10.1371/journal.ppat.1000974 (PMC2895647; doi:10.1371/journal.ppat.1000974)
Supplement: Table S1 — Designations and sources of wild-derived mice, cells and DNAs. (0.08 MB DOC) [file ppat.1000974.s001.doc]

Table S1. Designations and sources of wild-derived mice, cells and DNAs.

| **Subgenus** | **Species^a^** | **Previous species name or other designation** | **Geographic location** | **Type** | **Source** |
| --- | --- | --- | --- | --- | --- |
| *Coelomys* | *pahari*** |  |  | cells | Rodgers |
| *Pyromys* | *shortridgei** |  |  | cells | Rodgers |
|  | *saxicola*** |  | Mysore, India | mice | Potter |
| *Nannomys* | *minutoides*** |  |  | cells | Rodgers |
|  | *setulosus** |  | Nairobi, Kenya | cells | Rodgers |
|  | *tenellus*** |  | Kidepo, Uganda | DNA | d’Eustachio |
| *Mus* | *cervicolor cervicolor*** |  | Loei Province, Thailand | mice | Potter |
|  | *cookii*** |  | Tak Province, Thailand | mice | Potter |
|  | *caroli*** |  | Chonburi Province, Thailand | mice | Potter |
|  | *fragilicauda*** |  |  | cells | Hartley |
|  | *macedonicus*** |  |  | DNA | Elliott |
|  | *terricolor** | *dunni* | India | cells | Lander |
|  | *spicilegus*** | *hortulanus* | Halbturn, Austria | mice | Potter |
|  | *spicilegus** |  |  | cells | Rodgers |
|  | *spretus*** |  | Puerto Real, Spain | mice | Jackson Laboratory |
|  | *musculus castaneus* | CAST/N | Thailand | mice | Potter |
|  | *musculus castaneus*** | CAST/EiJ | Thailand | mice | Jackson Laboratory |
|  | *musculus castaneus* | CAST/Rp |  | mice | Roswell Park |
|  | *musculus castaneus** | CAS/Li |  | mice | Potter |
|  | *musculus molossinus*** |  | Kyushu, Japan | mice | Potter |
|  | *musculus molossinus* | MOLD/RkJ, MOLF/EiJ, MOLG/DnJ  MSM/Ms | various in Japan | DNA | Jackson Laboratory |
|  | *musculus*  *musculus* | Skive | Skive, Denmark | mice | Potter |
|  | *musculus musculus*** | CzI, CZECHI/EiJ | Morovia, Czechoslovakia | mice, DNA | Potter, Jackson Laboratory |
|  | *musculus musculus* | CzII, CZECHII/EiJ | Slovakia, Czechoslovakia | mice, DNA | Potter, Jackson Laboratory |
|  | *musculus musculus* | PWD/PhJ | Kunratice, Czech Republic | DNA | Jackson Laboratory |
|  | *musculus musculus* | PWK/PhJ | Lhotka, Czech Republic | DNA | Jackson Laboratory |
|  | *musculus musculus* | VEJ | Vejrumbro, Denmark | mice | Potter |
|  | *musculus domesticus*** | LW,LEWES/EiJ | Lewes,DE | mice, DNA | Potter, Jackson Laboratory |
|  | *musculus domesticus*** | CL(Centreville Lite) | Centreville, Maryland | mice | Potter |
|  | *musculus*  *domesticus* | WSA (Watkins Star) | Watkins Farm | mice | Potter |
|  | *musculus domesticus* | HF (Havens Farm) | Davidsonville, Maryland | mice | Potter |
|  | *musculus**,**,*** | CalWM, SC-1 cells, CALB/RkJ | Lake Casitas and Bouquet Canyon, California | mice | Rasheed, Potter, Jackson Laboratory |
|  | *musculus* | PERA/EiJ, PERC/EiJ | Rimac Valley, Peru | DNA | Jackson Laboratory |
|  | *musculus domesticus*** | JJD (J.J.Downs) | Ridgely, Maryland | mice | Potter |
|  | *musculus domesticus* | SF (Sanner’s Farm) | Davidsonville, Maryland | mice | Potter |
|  | *musculus domesticus* | *praetextus* (prae) |  | mice | Potter |
|  | *musculus domesticus** | *praetextus* (prae) |  | cells | Rodgers |
|  | *musculus domesticus* | *poschiavinus* (Posch-1) | Tirano, Italy | mice | Potter |
|  | *musculus domesticus* | *poschiavinus* (Posch-2) | Zalende, Switzerland | mice | Potter |

^a^Asterisks designate mice used to sequence full length mA3(*) or mA3 exons 2-4 (**). Other listed mice were analyzed by PCR for the presence of the LTR insertion.
