# Supplementary material for: Adaptive Evolution of Mus Apobec3 Includes Retroviral Insertion and Positive Selection at Two Clusters of Residues Flanking the Substrate Groove
Source: PLoS Pathog. 2010 Jul 1;6(7):e1000974. doi: 10.1371/journal.ppat.1000974 (PMC2895647; doi:10.1371/journal.ppat.1000974)
Supplement: Table S2 — PAML summary results for mA3 exons 2–4. (0.10 MB DOC) [file ppat.1000974.s002.doc]

Table S2. PAML summary results for mA3 exons 2-4.

1. Log likelihood scores and parameter estimates for the four models of variable dN/dS ratios among codons under the F3x4 model of codon frequencies for the complete set of exon 2-4 sequences on data-based phylogenetic tree 1.

| Model | Parameter Estimates | Sites with P(ω > 1) > 0.95 (Bayes Empirical Bayes) | lnL |
| --- | --- | --- | --- |
| M1:neutral | ω_0_ = 0 *f*_0_ = 0.605  ω_1_ = 1 *f*_1_ = 0.395  dN/dS for each branch = 0.4543  S = 1.8366 | NA | -2024.76 |
| M2: selection | ω_0_ = 0 *f*_0_ = 0.610  ω_1_ = 1 *f*_1_ = 0.225  ω_2_ = 4.391 *f*_2_ = 0.165  dN/dS for each branch = 1.0731  S = 1.9659 | 34 G 0.995  37 K 1.000  38 G 1.000 134 V 0.999  135 Q 1.000  136 D 0.995  183 L 0.982 | -1997.08 |
| M7:β distribution | *p* = 0.0247 *q* = 0.0214  dN/dS for each branch = 0.5229  S = 1.8091 | NA | -2028.42 |
| M8: β + positive selection | *p* = 1.2600 *q* = 2.1873  dN/dS for each branch = 1.1037  S = 1.9615  ω_1_ = 4.1679 *f*_1_ = 0.1945 | 34 G 0.999  37 K 1.000  38 G 1.000  134 V 0.999  135 Q 1.000  136 D 0.999  138 E 0.975  183 L 0.991 | -1997.53 |

2. Log likelihood scores and parameter estimates for the four models of variable dN/dS ratios among codons under the F61 (codon table) model of codon frequencies for the complete set of exon 2-3-4 sequences on data-based phylogenetic tree 1.

| Model | Parameter Estimates | Sites with P(ω > 1) > 0.95 (Bayes Empirical Bayes) | lnL |
| --- | --- | --- | --- |
| M1:neutral | ω_0_ = 0 *f*_0_ = 0.610  ω_1_ = 1 *f*_1_ = 0.390  dN/dS for each branch = 0.454  S = 1.8656 | NA | -2036.94 |
| M2: selection | ω_0_ = 0 *f*_0_ = 0.608  ω_1_ = 1 *f*_1_ = 0.230  ω_2_ = 4.542 *f*_2_ = 0.162  dN/dS for each branch = 1.0977  S = 1.9987 | 34 G 0.994  37 K 0.999  38 G 1.000 134 V 0.998  135 Q 1.000  136 D 0.997  175 R 0.960  183 L 0.963 | -2009.86 |
| M7:β distribution | *p* = 0.1347 *q* = 0.1317  dN/dS for each branch = 0.5057  S = 1.8508 | NA | -2041.14 |
| M8: β + positive selection | *p* = 1.2581 *q* = 2.0005  dN/dS for each branch = 1.1235  S = 1.9947  ω_1_ = 4.3224 *f*_1_ = 0.1876 | 25 T 0.967  34 G 0.998  37 K 1.000  38 G 1.000  128 S 0.951  134 V 0.999  135 Q 1.000  136 D 0.999  138 E 0.973  175 R 0.973  183 L 0.976 | -2010.26 |

3. Likelihood ratio test statistics for models of variable selective pressure among codons calculated on data-based phylogenetic tree 1.

|  | 2(lnL_1_-lnL_0_) | df | P value |
| --- | --- | --- | --- |
| F3x4 |  |  |  |
| M1 vs. M2 | 55.3559 | 2 | 9.54 x 10^-13^ |
| M7 vs. M8 | 61.7989 | 2 | 3.81x10^-14^ |
| F61 |  |  |  |
| M1 vs. M2 | 54.1613 | 2 | 1.74x10^-12^ |
| M7 vs. M8 | 61.7770 | 2 | 3.85x10^-14^ |

4. Likelihood ratio test statistics for models of variable selective pressure along lineages for data-based phylogenetic tree 1.

|  | lnL | 2(lnL_1_-lnL_0_) | df | P value |
| --- | --- | --- | --- | --- |
| Model 0 (same dN/dS for all branches) | -2036.8905 |  |  |  |
| Model 1 (different dN/dS for each branch) | -2064.9501 | 56.1191 | 49 | 0.2255 |

For this LRT the degrees of freedom is one less than the number of branches in the phylogeny. For the analysis of all Mus lineages there were 50 branches.

5. Log likelihood scores and parameter estimates for the four models of variable dN/dS ratios among codons under the F3x4 model of codon frequencies for the complete set of exon 2-3-4 sequences on taxonomy-based phylogenetic tree 2.

| Model | Parameter Estimates | Sites with P(ω > 1) > 0.95 (Bayes Empirical Bayes) | lnL |
| --- | --- | --- | --- |
| M1:neutral | ω_0_ = 0 *f*_0_ = 0.619  ω_1_ = 1 *f*_1_ = 0.381  dN/dS for each branch = 0. 4425  S = 1.8701 | NA | -2044.21 |
| M2: selection | ω_0_ = 0 *f*_0_ = 0.641  ω_1_ = 1 *f*_1_ = 0.188  ω_2_ = 4.575 *f*_2_ = 0.171  dN/dS for each branch = 1.1162  S = 1.9986 | 34 G 0.996  37 K 1.000  38 G 1.000  60 H 0.957 134 V 0.999  135 Q 1.000  136 D 0.996  138 E 0.956  139 T 0.950  183 L 0.986 | -2012.74 |
| M7:β distribution | *p* = 0.1173 *q* = 0.1179  dN/dS for each branch = 0.4987  S = 1.8622 | NA | -2048.84 |
| M8: β + positive selection | *p* = 1.33249 *q* = 2.25419  dN/dS for each branch = 1.1285  S = 1.99413  ω_1_ = 4.435 *f*_1_ = 0.1866 | 34 G 0.999  37 K 1.000  38 G 1.000  60 H 0.981 134 V 1.000  135 Q 1.000  136 D 0.999  138 E 0.977  139 T 0.974  181 R 0.969  183 L 0.992 | -2013.01 |

6. Log likelihood scores and parameter estimates for the four models of variable dN/dS ratios among codons under the F61 (codon table) model of codon frequencies for the complete set of exon 2-3-4 sequences on taxonomy-based phylogenetic tree 2.

| Model | Parameter Estimates | Sites with P(ω > 1) > 0.95 (Bayes Empirical Bayes) | lnL |
| --- | --- | --- | --- |
| M1:neutral | ω_0_ = 0 *f*_0_ = 0.625  ω_1_ = 1 *f*_1_ = 0.375  dN/dS for each branch = 0.442  S = 1.8906 | NA | -2052.55 |
| M2: selection | ω_0_ = 0 *f*_0_ = 0.621  ω_1_ = 1 *f*_1_ = 0.219  ω_2_ = 4.816 *f*_2_ = 0.159  dN/dS for each branch = 1.1310  S = 2.0265 | 34 G 0.995  37 K 0.999  38 G 1.000  60 H 0.979  134 V 0.998  135 Q 1.000  136 D 0.996  138 E 0.954  139 T 0.961  175 R 0.967  183 L 0.968 | -2021.89 |
| M7:β distribution | *p* = 0. 1405 *q* = 0.1424  dN/dS for each branch = 0.4967  S = 1.8771 | NA | -2057.30 |
| M8: β + positive selection | *p* = 1.1283 *q* = 1.6701  dN/dS for each branch = 1.1447  S = 2.0229  ω_1_ = 4.6689 *f*_1_ = 0.1740 | 25 T 0.967  34 G 0.998  37 K 1.000  38 G 1.000  60 H 0.991  113 V 0.966 134 V 0.999  135 Q 1.000  136 D 0.999  138 E 0.976  139 T 0.980  175 R 0.976  183 L 0.977 | -2022.17 |

7. Likelihood ratio test statistics for models of variable selective pressure among codons calculated on taxonomy-based phylogenetic tree 2.

|  | 2(lnL_1_-lnL_0_) | Df | P value |
| --- | --- | --- | --- |
| F3x4 |  |  |  |
| M1 vs. M2 | 62.9343 | 2 | 2.16 x 10^-14^ |
| M7 vs. M8 | 71.6706 | 2 | 2.74 x 10^-16^ |
| F61 |  |  |  |
| M1 vs. M2 | 61.3244 | 2 | 4.83 x 10^-14^ |
| M7 vs. M8 | 70.2655 | 2 | 5.52 x 10^-16^ |

8. Likelihood ratio test statistics for models of variable selective pressure along lineages for taxonomy-based phylogenetic tree 2.

|  | lnL | 2(lnL_1_-lnL_0_) | df | P value |
| --- | --- | --- | --- | --- |
| Model 0 (same dN/dS for all branches) | -2059.0472 |  |  |  |
| Model 1 (different dN/dS for each branch) | -2087.3800 | 56.6657 | 48 | 0.1831 |

For this LRT the degrees of freedom is one less than the number of branches in the phylogeny. For the analysis of all Mus lineages there were 49 branches.
