# Supplementary material for: Adaptive Evolution of Mus Apobec3 Includes Retroviral Insertion and Positive Selection at Two Clusters of Residues Flanking the Substrate Groove
Source: PLoS Pathog. 2010 Jul 1;6(7):e1000974. doi: 10.1371/journal.ppat.1000974 (PMC2895647; doi:10.1371/journal.ppat.1000974)
Supplement: Table S3 — PAML summary results for Apobec3 full-length sequence. (0.10 MB DOC) [file ppat.1000974.s003.doc]

Table S3. PAML summary results for *Apobec3* full-length sequence.

1. Log likelihood scores and parameter estimates for the four models of variable dN/dS ratios among codons under the F3x4 model of codon frequencies for the complete set of full-length sequences on data-based phylogenetic tree 1.

| Model | Parameter Estimates | Sites with P(ω > 1) > 0.95 (Bayes Empirical Bayes) | lnL |
| --- | --- | --- | --- |
| M1:neutral | ω_0_ = 0 *f*_0_ = 0.425  ω_1_ = 1 *f*_1_ = 0.575  dN/dS for each branch = 0.5754  S = 0.5882 | NA | -2457.83 |
| M2: selection | ω_0_ = 0 *f*_0_ = 0.380  ω_1_ = 1 *f*_1_ = 0.533  ω_2_ = 6.197  *f*_2_ = 0.086  dN/dS for each branch = 1.0677  S = 0.6339 | 38 G 0.997  135 Q 0.997 | -2445.63 |
| M7:β distribution | *p* = 0.0075  *q* = 0.0050  dN/dS for each branch = 0.6000  S = 0.5898 | NA | -2457.90 |
| M8: β + positive selection | *p* = 0.0073  *q* = 0.0050  ω_1_ = 6.4155  *f*_1_ = 0.0828  dN/dS for each branch = 1.1037  S = 0.6349 | 38 G 0.999  135 Q 0.998  138 E 0.957  142 N 0.957  183 L 0.969 | -2445.64 |

2. Log likelihood scores and parameter estimates for the four models of variable dN/dS ratios among codons under the F61 (codon table) model of codon frequencies for the complete set of full-length sequences on data-based phylogenetic tree 1.

| Model | Parameter Estimates | Sites with P(ω > 1) > 0.95 (Bayes Empirical Bayes) | lnL |
| --- | --- | --- | --- |
| M1:neutral | ω_0_ = 0 *f*_0_ = 0.418  ω_1_ = 1 *f*_1_ = 0.582  dN/dS for each branch = 0.5821  S = 0.5931 | NA | -2410.16 |
| M2: selection | ω_0_ = 0 *f*_0_ = 0.377  ω_1_ = 1 *f*_1_ = 0.519  ω_2_ = 6.010  *f*_2_ = 0.104  dN/dS for each branch = 1.1430  S = 0.6369 | 38 G 0.998  135 Q 0.997  183 L 0.956 | -2396.96 |
| M7:β distribution | *p* = 0.0159  *q* = 0.0141  dN/dS for each branch = 0.5057  S = 0.5864 | NA | -2411.02 |
| M8: β + positive selection | *p* = 0.0075  *q* = 0.0050  ω_1_ = 6.2584  *f*_1_ = 0.0986dN/dS for each branch = 1.1235  S = 0.6377 | 38 G 0.999  135 Q 0.999  138 E 0.962  142 N 0.965  183 L 0.975  201 D 0.962  316 P 0.962 | -2396.98 |

3. Likelihood ratio test statistics for models of variable selective pressure among codons calculated on data-based phylogenetic tree 1.

|  | 2(lnL_1_-lnL_0_) | df | P value |
| --- | --- | --- | --- |
| F3x4 |  |  |  |
| M1 vs. M2 | 24.3956 | 2 | 5.04 x 10^-6^ |
| M7 vs. M8 | 24.5069 | 2 | 4.77x10^-6^ |
| F61 |  |  |  |
| M1 vs. M2 | 26.3925 | 2 | 1.86x10^-6^ |
| M7 vs. M8 | 28.0850 | 2 | 7.97x10^-7^ |

4. Likelihood ratio test statistics for models of variable selective pressure along lineages for data-based phylogenetic tree 1.

|  | lnL | 2(lnL_1_-lnL_0_) | df | P value |
| --- | --- | --- | --- | --- |
| Model 0 (same dN/dS for all branches) | -2463.0383 |  |  |  |
| Model 1 (different dN/dS for each branch) | -2469.4882 | 12.8997 | 12 | 0.3764 |

For this LRT the degrees of freedom is one less than the number of branches in the phylogeny. For the analysis of all *Mus* lineages there were 13 branches.

5. Log likelihood scores and parameter estimates for the four models of variable dN/dS ratios among codons under the F3x4 model of codon frequencies for the complete set of full-length sequences on taxonomy-based phylogenetic tree 2.

| Model | Parameter Estimates | Sites with P(ω > 1) > 0.95 (Bayes Empirical Bayes) | lnL |
| --- | --- | --- | --- |
| M1:neutral | ω_0_ = 0 *f*_0_ = 0.462  ω_1_ = 1 *f*_1_ = 0.538  dN/dS for each branch = 0.5379  S = 0.6181 | NA | -2504.10 |
| M2: selection | ω_0_ = 0 *f*_0_ = 0.408  ω_1_ = 1 *f*_1_ = 0.493  ω_2_ = 6.529  *f*_2_ = 0.099  dN/dS for each branch = 1.1406  S = 0.6713 | 38 G 0.997  135 Q 0.998  138 E 0.951  183 L 0.963  201 D 0.979  273 T 0.956  371 R 0.979 | -2486.83 |
| M7:β distribution | *p* = 0.0055  *q* = 0.0054  dN/dS for each branch = 0.5000  S = 0.6129 | NA | -2504.32 |
| M8: β + positive selection | *p* = 0.0304  *q* = 0.0260  ω_1_ = 6.367  *f*_1_ = 0.1037  dN/dS for each branch = 1.1367  S = 0.6706 | 37 K 0.965  38 G 0.999  113 V 0.967  135 Q 0.999  138 E 0.972  142 N 0.970  183 L 0.978  201 D 0.990  273 T 0.975  316 P 0.956  371 R 0.990 | -2486.84 |

6. Log likelihood scores and parameter estimates for the four models of variable dN/dS ratios among codons under the F61 (codon table) model of codon frequencies for the complete set of full-length sequences on taxonomy-based phylogenetic tree 2.

| Model | Parameter Estimates | Sites with P(ω > 1) > 0.95 (Bayes Empirical Bayes) | lnL |
| --- | --- | --- | --- |
| M1:neutral | ω_0_ = 0 *f*_0_ = 0.457  ω_1_ = 1 *f*_1_ = 0.543  dN/dS for each branch = 0.5430  S = 0.6237 | NA | -2456.99 |
| M2: selection | ω_0_ = 0 *f*_0_ = 0.406  ω_1_ = 1 *f*_1_ = 0.477  ω_2_ = 6.369  *f*_2_ = 0.116  dN/dS for each branch = 1.2184  S = 0.6734 | 38 G 0.998  113 V 0.955  135 Q 0.999  138 E 0.956  142 N 0.955  183 L 0.969  201 D 0.986  273 T 0.964  316 P 0.951  371 R 0.985 | -2438.35 |
| M7:β distribution | *p* = 0.0052  *q* = 0.0050  dN/dS for each branch = 0.5000  S = 0.6187 | NA | -2457.27 |
| M8: β + positive selection | *p* = 0.0281  *q* = 0.0244  ω_1_ = 6.229  *f*_1_ = 0.1211  dN/dS for each branch = 1.2152  S = 0.6729 | 34 G 0.954  37 K 0.968  38 G 0.999  113 V 0.974  128 S 0.950  135 Q 0.999  138 E 0.974  139 T 0.952  142 N 0.974  183 L 0.982  201 D 0.993  273 T 0.979  316 P 0.971  371 R 0.993 | -2438.36 |

7. Likelihood ratio test statistics for models of variable selective pressure among codons calculated on taxonomy-based phylogenetic tree 2.

|  | 2(lnL_1_-lnL_0_) | df | P value |
| --- | --- | --- | --- |
| F3x4 |  |  |  |
| M1 vs. M2 | 34.5373 | 2 | 3.16 x 10^-8^ |
| M7 vs. M8 | 34.9484 | 2 | 2.57 x 10^-8^ |
| F61 |  |  |  |
| M1 vs. M2 | 37.2858 | 2 | 8.01 x 10^-9^ |
| M7 vs. M8 | 37.8064 | 2 | 6.17 x 10^-9^ |

8. Likelihood ratio test statistics for models of variable selective pressure along lineages for taxonomy-based phylogenetic tree 2.

|  | lnL | 2(lnL_1_-lnL_0_) | df | P value |
| --- | --- | --- | --- | --- |
| Model 0 (same dN/dS for all branches) | -2512.7034 |  |  |  |
| Model 1 (different dN/dS for each branch) | -2520.1403 | 14.8738 | 12 | 0.2484 |

For this LRT the degrees of freedom is one less than the number of branches in the phylogeny. For the analysis of all *Mus* lineages there were 13 branches.
