# Supplementary figures and images for: Adaptive Evolution of Mus Apobec3 Includes Retroviral Insertion and Positive Selection at Two Clusters of Residues Flanking the Substrate Groove
Source: PLoS Pathog. 2010 Jul 1;6(7):e1000974. doi: 10.1371/journal.ppat.1000974 (PMC2895647; doi:10.1371/journal.ppat.1000974)

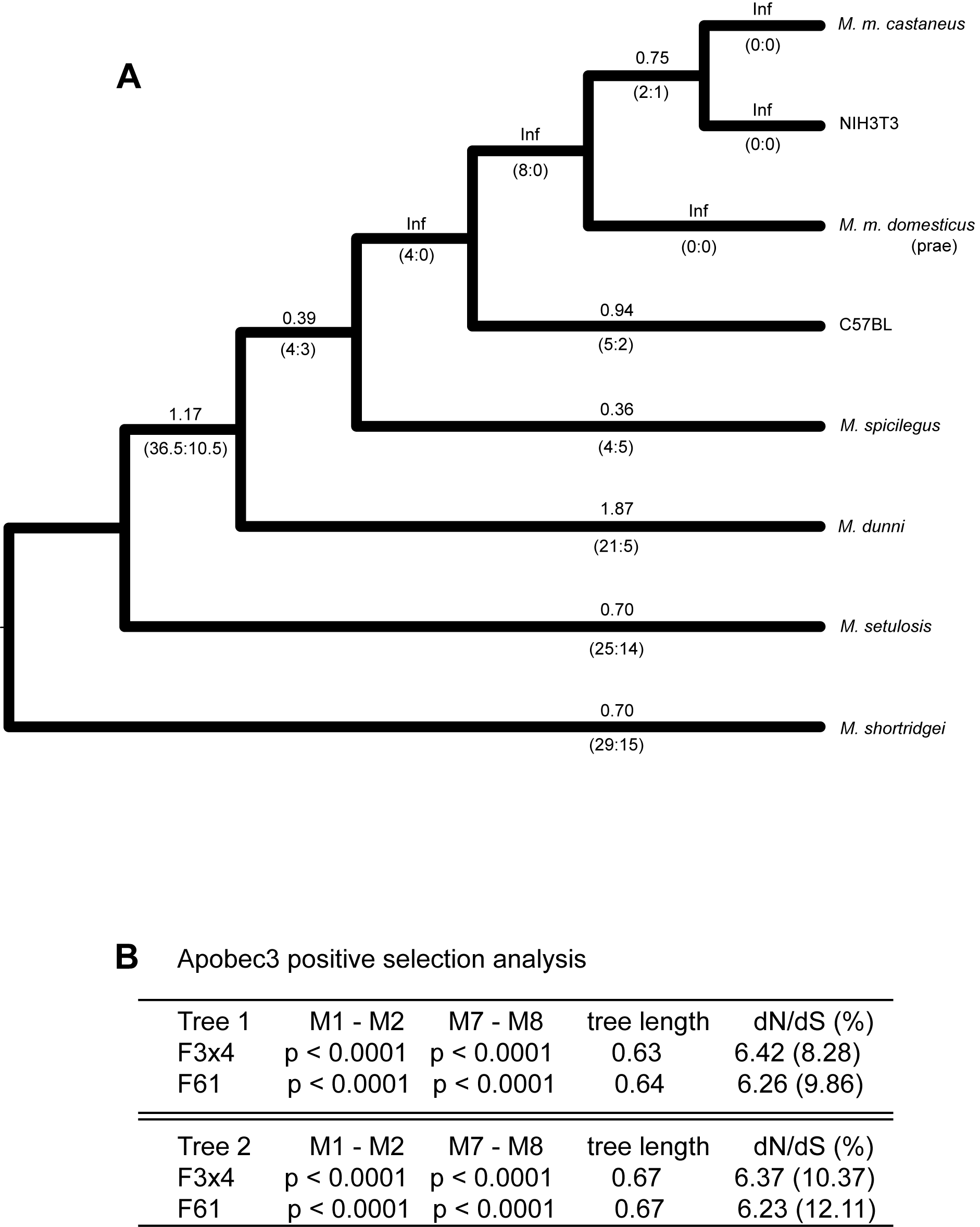

Supplement: Figure S1 — Phylogenetic tree and likelihood ratio tests for the mA3 full length sequence. A) Data-derived cladogram showing branch values of dN/dS calculated using the free-ratio model of PAML, with the number of replacement and synonymous changes in parentheses. When dS = 0, dN/dS is infinite (Inf). dN/dS>1 suggests positive selection along that lineage. B) Likelihood ratio tests were used to tests for positive selection. Neutral models (M1, M7) were compared with selection models (M2, M8) using two different models of codon frequency (F3X4 or F61). P values <0.0001 provide strong evidence of positive selection. Tree 1 is the data-derived tree and tree 2 is the taxonomy-derived tree. Tree length is the average number of substitutions per codon along all branches. dN/dS ratio is given for the codons under selection, along with the % of codons in this category. (0.47 MB TIF) [file ppat.1000974.s004.tif]
