# Supplementary material for: Adaptive Evolution of Mus Apobec3 Includes Retroviral Insertion and Positive Selection at Two Clusters of Residues Flanking the Substrate Groove
Source: PLoS Pathog. 2010 Jul 1;6(7):e1000974. doi: 10.1371/journal.ppat.1000974 (PMC2895647; doi:10.1371/journal.ppat.1000974)
Supplement: Text S1 — Alignment of full-length mA3 sequences from mice listed in Table S1. The codons under positive selection by PAML are boxed, with red boxes indicating codons under very strong selection (P>.99). Green fill marks codons that distinguish C57BL and BALB/c. Selection analysis by maximum likelihood is limited to sites typed in all DNAs and excludes 72 bp at the 5′ end, 33 bp at the 3′ end and the 99 bp exon 5. The C57BL mA3 sequence was from GenBank (No. NM_030255). The sequence derived from NIH 3T3 was used in place of BALB/c as all clones from BALB 3T3 cells lacked exons 2 and 5, and the NIH 3T3 sequence was otherwise identical to the published BALB/c sequence (GenBank No. EDL04624). (0.07 MB DOC) [file ppat.1000974.s005.doc]

C57BL ACATTCAAGTTCCACTTTAAGAACCTAGGCTATGCCAAAGGCCGGAAAGATACCTTCTTG

*M.m. castaneus*  ACATTCAAATTCCACTTTAAGAACCTACGCTATGCCATAGACCGGAAAGATACCTTCTTG

*M.dunni*  ACATTCAAGTTCCACTTTGAGAACCTACCCTTTGCCAAAAAACGGAAAGATACCTTCTTG

NIH3T3 ACATTCAAATTCCACTTTAAGAACCTACGCTATGCCATAGACCGGAAAGATACCTTCTTG

*M.m. domesticus* (prae) ACATTCAAATTCCACTTTAAGAACCTACGCTATGCCAAAGGCCGGAAAGATACCTTCTTG

*M.spicilegus*  ACATTCAAATTCCACTTCAAGAACCTACGCTATGCCAAAGACCGGAAAGATACCTTCTTG

*M.setulosus*  ACATTCAACTTCAACTTTGAGAACCTATGCTATGCCGAAGGCCGAAAAAATACCTTCTTG

*M.shortridgei*  AAATTCGACTTCCACTTTAAGAACCTATGCTATGCCAAAGACCGGAAAGATACCTTCTTG

C57BL TGCTATGAAGTGACTAGAAAGGACTGCGATTCACCCGTCTCCCTTCACCATGGGGTCTTT

*M.m. castaneus*  TGCTATGAAGTGACTAGAAAGGACTGCGATTCACCCGTCTCCCTTCACCATGGGGTCTTT

*M.dunni*  TGCTATGAAGTGACTAGAAAGGACTGCGATTCACCCGTCTCCCTTCACCATGGGGTCTTT

NIH3T3 TGCTATGAAGTGACTAGAAAGGACTGCGATTCACCCGTCTCCCTTCACCATGGGGTCTTT

*M.m. domesticus* (prae) TGCTATGAAGTGACTAGAAAGGACTGCGATTCACCCGTCTCCCTTCACCATGGGGTCTTT

*M.spicilegus*  TGCTATGAAGTGACTAGAAAGGACTGCGATTCACCCGTCTCCCTTCACCATGGGGTCTTT

*M.setulosus*  TGCTATGAAGTGACTAGAAAGGACTGTGATTCACCCGTTTCCCTTTGCCATGGAGTCTTT

*M.shortridgei*  TGCTATGAAGTGACTAGAAAGGACTGCGATTCACCCAACTCCCTTTGCCATGGGGTCTTT

C57BL AAGAACAAGGACAACATCCACGCTGAAATCTGCTTTTTATACTGGTTCCATGACAAAGTA

*M.m. castaneus*  AAGAACAAGGACAACATCCACGCTGAAATCTGCTTTTTATACTGGTTCCATGACAAAGTA

*M.dunni*  AAGAACAAGGACAACATCCACGCTGAAATCTGCTTTTTATACTGGTTCCATGACAAAGTA

NIH3T3 AAGAACAAGGACAACATCCACGCTGAAATCTGCTTTTTATACTGGTTCCATGACAAAGTA

*M.m. domesticus* (prae) AAGAACAAGGACAACATCCACGCTGAAATCTGCTTTTTATACTGGTTCCATGACAAAGTA

*M.spicilegus*  AAGAACAAGGACAACATCCACGCTGAAATCTGCTTTTTATACTGGTTCCATGACAAAGTA

*M.setulosus*  AAGAACAAGGGCAGCATCCACGCTGAAATCTGCTTTTTATACTGGTTCCATGACAAAGTA

*M.shortridgei*  AAGAACAAGGGCAACACCCACGCTGAAATCTGCTTTTTATACTGGTTCCATGACAAAGTA

C57BL CTGAAAGTGCTGTCTCCGAGAGAAGAGTTCAAGATCACCTGGTATATGTCCTGGAGCCCC

*M.m. castaneus*  CTGAAAGTGCTGTCTCCGAGAGAAGAGTTCAAGATCACCTGGTATATGTCCTGGAGCCCC

*M.dunni*  CTGAAAGTGCTATCTCCGAGGGAAGAGTTCAAGATCACCTGGTATATGTCCTGGAGCCCC

NIH3T3 CTGAAAGTGCTGTCTCCGAGAGAAGAGTTCAAGATCACCTGGTATATGTCCTGGAGCCCC

*M.m. domesticus* (prae) CTGAAAGTGCTGTCTCCGAGAGAAGAGTTCAAGATCACCTGGTATATGTCCTGGAGCCCC

*M.spicilegus*  TTGAAAGTGCTGTCTCCGAGAGAAGAGTTCAAGATCACCTGGTATATGTCCTGGAGCCCC

*M.setulosus*  CTGAAAGTGCTGACTCAGAGGGAAGGGTTCAAGGTCACCTGGTATATGTCCTGGAGCCCC

*M.shortridgei*  CTAAAAGTGCTGTCTCTGAGGGAAGAGTTCAAGGTCACCTGGTACATGTCCTGGAGCCCC

C57BL TGTTTCGAATGTGCAGAGCAGATAGTAAGGTTCCTGGCTACACACCACAACCTGAGCCTG

*M.m. castaneus*  TGTTTCGAATGTGCAGAGCAGGTACTAAGGTTCCTGGCTACACACCACAACCTGAGCCTG

*M.dunni*  TGTTTCGAATGTGCAGAGCAGGTAGTAAGGTTCCTGGCCACACACCACAACCTGAGCCTG

NIH3T3 TGTTTCGAATGTGCAGAGCAGGTACTAAGGTTCCTGGCTACACACCACAACCTGAGCCTG

*M.m. domesticus* (prae) TGTTTCGAATGTGCAGAGCAGGTACTAAGGTTCCTGGCTACACACCACAACCTGAGCCTG

*M.spicilegus*  TGTTTCGAATGTGCAGAGCAGGTAGTAAGGTTCCTGGCTACACACCACAACCTGAGCCTG

*M.setulosus*  TGTTTTGAATGTGCAGAGCAGGTAGTCAGGTTCCTGGCCACACACCACAACCTGAACCTG

*M.shortridgei*  TGCTTCGAATGCGCAGAGCAGGTAGCCAGGTTCCTGGCCACACACCACAACCTGAGCCTG

C57BL GACATCTTCAGCTCCCGCCTCTACAACGTACAGGACCCAGAAACCCAGCAGAATCTTTGC

*M.m. castaneus* GACATCTTCAGCTCCCGCCTCTACAACATACGGGACCCAGAAAACCAGCAGAATCTTTGC

*M.dunni* GACATCTTCTTCTCCCGCCTCTACAACATAAGGAACCCAGAAAACCAGCAGAATCTTTGC

NIH3T3 GACATCTTCAGCTCCCGCCTCTACAACATACGGGACCCAGAAAACCAGCAGAATCTTTGC

*M.m. domesticus* (prae) GACATCTTCAGCTCCCGCCTCTACAACATACGGGACCCAGAAAACCAGCAGAATCTTTGC

*M.spicilegus* GACATCTTCATCTCCCGCCTCTACAACATATGGGACCCAAAAACCCAGCAGAATCTTTGC

*M.setulosus*  ACCATCTTCAGCTCCCGCCTCTACAATGTATCAGACCCGGACACCCAGCAGAAACTTTGC

*M.shortridgei*  GCCATCTTCAGCTCCCGCCTCTACAACATAAGGGACCCGAAGACCGGGCAGGGACTTTGC

C57BL AGGCTGGTTCAGGAAGGAGCCCAGGTGGCTGCCATGGACCTATACGAATTTAAAAAGTGT

*M.m. castaneus*  AGGCTGGTTCAGGAAGGAGCCCAGGTGGCTGCCATGGACCTATACGAATTTAAAAAGTGT

*M.dunni*  AGGCTGGTTCTGGAAGGAGCCCAGGTGGCTGCCATGGACCTATACGAATTTGAAGAGTGT

NIH3T3 AGGCTGGTTCAGGAAGGAGCCCAGGTGGCTGCCATGGACCTATACGAATTTAAAAAGTGT

*M.m. domesticus* (prae) AGGCTGGTTCAGGAAGGAGCCCAGGTGGCTGCCATGGACCTATACGAATTTAAAAAGTGT

*M.spicilegus*  AGGCTGGTTCAGGAAGGAGCCCAGGTGGCTGCCATGGACCTATACGAATTTAAAAAGTGT

*M.setulosus*  AGGCTGGTTCAGGAAGGAGCCCAGGTGGCTGTCATGGACCTATCCGAATTTAAAAAGTGT

*M.shortridgei*  AGGCTGGTTCAGGAAGGAGCCCAGGTGGCTGCCATGGGCCTACCCGAATTTGAAAAGTGT

C57BL TGGAAGAAGTTTGTGGACAATGGTGGCAGGCGATTCAGGCCTTGGAAAAGACTGCTTACA

*M.m. castaneus*  TGGAAGAAGTTTGTGGACAATGGCGGCAGGCGATTCAGGCCTTGGAAAAAACTGCTTACA

*M.dunni*  TGGAAGAAGTTTGTGGACAATGGCGGCAGACGATTCAGGCCTTGGAAAAGACTGCTTACA

NIH3T3 TGGAAGAAGTTTGTGGACAATGGCGGCAGGCGATTCAGGCCTTGGAAAAAACTGCTTACA

*M.m. domesticus* (prae) TGGAAGAAGTTTGTGGACAATGGCGGCAGGCGATTCAGGCCTTGGAAAAAACTGCTTACA

*M.spicilegus*  TGGAAGAAGTTTGTGGACAACGGCGGCAGGCGATTCAGGCCTTGGAAAAGACTGCTTACA

*M.setulosus*  TGGGAGAAGTTTGTGGACAATGACGGCCAGCAATTCAGGCCTTGGAAGAGACTGCATACC

*M.shortridgei*  TGGAAGAAGTTTGTGGACAATGACGGCCAGCCATTCATGCCTTGGAAGAGACTGTGTACA

C57BL AATTTTAGATACCAGGATTCTAAGCTTCAGGAGATTCTGAGGCGAATGGACCCGCTAAGT

*M.m. castaneus*  AATTTTAGATACCAGGATTCTAAGCTTCAGGAGATTCTGAGACGAGTGCACCTGCTAAGT

*M.dunni*  AATTTTAGATACCAGGATTCTAAGCTTCAGGAGATTCTGAGGCGAATGAGCCTGCTAAGT

NIH3T3 AATTTTAGATACCAGGATTCTAAGCTTCAGGAGATTCTGAGACGAGTGCACCTGCTAAGT

*M.m. domesticus* (prae) AATTTTAGATACCAGGATTCTAAGCTTCAGGAGATTCTGAGGCGAGTGCACCTGCTAAGT

*M.spicilegus*  AATTTTAGATACCAGGATTCTAAGCTTCAGGAGATTCTGAGGCGAATGGGCCTGCTAAGT

*M.setulosus*  AATTTTAGATATCAGAATTCTAAGCTTCAGGAGATTCTGAGCCGAATGAGCCTGCTAAGA

*M.shortridgei*  AATTTTAGATACCAGAATTCTAAGCTTCAGGAGATTCTGAGCCAAATGAGCCTGCTAAGA

C57BL GAAGAGGAATTTTACTCGCAGTTTTACAACCAACGA---GTCAAG------CATCTCTGC

*M.m. castaneus*  GAAGAGGAATTTTACTCGCAGTTTTACAACCAACGA---GTCAAG------CATCTCTGC

*M.dunni*  GAAGAGGAATTTTACTCGCAGTTTTACAACCTACGA---GTCAAG------CATCTCTGC

NIH3T3 GAAGAGGAATTTTACTCGCAGTTTTACAACCAACGA---GTCAAG------CATCTCTGC

*M.m. domesticus* (prae) GAAGAGGAATTTTACTCGCAGTTTTACAACCAACGA---GTCAAG------CATCTCTGC

*M.spicilegus*  GAAGAGGAATTTTACTCGCAGTTTTACAACCAACGA---GTCAAG------CATCTCTGC

*M.setulosus*  GAAGAGGTTTTTTACTTGCAATTTCACAACGGCCACAAGGTCCAGCTAGTCCAGCATCAC

*M.shortridgei*  GAAGAGGTGTTTTATTTACAATTTCACAACAGCCGAAAGGTCCAGCCAGTCCAGCATCGC

C57BL TACTACCACCGCATGAAGCCCTATCTATGCTACCAGCTGGAGCAGTTCAATGGCCAAGCG

*M.m. castaneus*  TACTACCACGGCATGAAGCCCTATCTATGCTACCAGCTGGAGCAGTTCAATGGCCAAGCG

*M.dunni*  TACTACCACCGCATGAAACCCTATCTATTCTACCAGCTGGAGTGGTTCAATGGCCAAGAG

NIH3T3 TACTACCACGGCATGAAGCCCTATCTATGCTACCAGCTGGAGCAGTTCAATGGCCAAGCG

*M.m. domesticus* (prae) TACTACCACGGCATGAAGCCCTATCTATGCTACCAGCTGGAGCAGTTCAATGGCCAAGCG

*M.spicilegus*  TACTACCACCGCATGAAGCCCTATCTATGCTACCAGTTGGAGCAGTTCAATGGCCAAGCG

*M.setulosus*  TGCTACTTCCGCAGAAAGCCCTATCTATGCTACCGGCTGGAGCAGTCCAATGGCCAAGAG

*M.shortridgei*  TGCTACTACCGCAGGAAGCCCTATCTATGCTACCGGCTGGAGCAGTCCAATGGCCAAGAG

C57BL CCACTCAAAGGCTGCCTGCTAAGCGAGAAAGGCAAACAGCATGCAGAAATCCTCTTCCTT

*M.m. castaneus*  CCACTCAAAGGCTGCCTGCTAAGCGAGAAAGGCAAACAGCATGCAGAAATCCTCTTCCTT

*M.dunni*  CCACTCAAAGGCTGCCTGCTAAGCGAGAAAGGCAAACAGCATGCAGAAATCCTCTTCCTT

NIH3T3 CCACTCAAAGGCTGCCTGCTAAGCGAGAAAGGCAAACAGCATGCAGAAATCCTCTTCCTT

*M.m. domesticus* (prae) CCACTCAAAGGCTGCCTGCTAAGCGAGAAAGGCAAACAGCATGCAGAAATCCTCTTCCTT

*M.spicilegus*  CCACTCAAAGGCTGCCTGCTAAGCGAGAAAGGCAAACAGCATGCAGAAATCCTCTTCCTT

*M.setulosus*  CTGCTCAAAGACTGCCTGCTGAGCAAGAAAGGTAAACAGCATGCAGAAATCCTCTTCCTT

*M.shortridgei*  CTGCTCAAAGGCTGGCTGCTAAGCAAGAAAGGAAAACAACATGCAGAAATCCTCTTCCTG

C57BL GATAAGATTCGGTCCATGGAGCTGAGCCAAGTGACAATCACCTGCTACCTCACCTGGAGC

*M.m. castaneus*  GATAAGATTCGGTCCATGGAGCTGAGCCAAGTGATAATCACCTGCTACCTCACCTGGAGC

*M.dunni*  AATAAGATTCGGTCCATGGAGCTGAGCCAAGTGACAATCACCTGCTACCTCACCTGGAGC

NIH3T3 GATAAGATTCGGTCCATGGAGCTGAGCCAAGTGATAATCACCTGCTACCTCACCTGGAGC

*M.m. domesticus* (prae) GATAAGATTCGGTCCATGGAGCTGAGCCAAGTGATAATCACCTGCTACCTCACCTGGAGC

*M.spicilegus*  GATAAGATTCGGTCCATGGAGCTGAGCCAAGTGACAATCACCTGCTACCTCACCTGGAGC

*M.setulosus*  GATAAGATTCGGTCCATGGAGCTGAGCCAAGTGAGAATCACCTGCTACCTCACCTGGAGC

*M.shortridgei*  GAGAAGATTCGGTCCATGGAGCTGAGTCAAATGAGAATCACCTGCTACCTCACTTGGAGC

C57BL CCCTGCCCAAACTGTGCCTGGCAACTGGCGGCATTCAAAAGGGATCGTCCAGATCTAATT

*M.m. castaneus*  CCCTGCCCAAACTGTGCCTGGCAACTGGCGGCATTCAAAAGGGATCGTCCAGATCTAATT

*M.dunni*  CCCTGCCCAAACTGTGCCTGGCAACTGGCGGCATTCAAAAAGGATCGCCCAGATCTAATT

NIH3T3 CCCTGCCCAAACTGTGCCTGGCAACTGGCGGCATTCAAAAGGGATCGTCCAGATCTAATT

*M.m. domesticus* (prae) CCCTGCCCAAACTGTGCCTGGCAACTGGCGGCATTCAAAAGGGATCGTCCAGATCTAATT

*M.spicilegus*  CCCTGCCCAAACTGTGCCTGGCAACTGGCAGCATTCAAAAGGGATCGTCCAGATCTAATT

*M.setulosus*  CCCTGCCCAAACTGTGCCTGGCAACTGGAGACATTCAAAAAGGATCGTCCAGATCTAATT

*M.shortridgei*  CCCTGCCCAAACTGTGCCTGGCAACTGGCGGCATTCCAAAAGGATCGCCCAGATCTAATT

C57BL CTGCATATCTACACCTCCCGCCTGTATTTCCACTGGAAGAGGCCCTTCCAGAAGGGGCTG

*M.m. castaneus*  CTGCATATCTACACCTCCCGCCTGTATTTCCACTGGAAGAGGCCCTTCCAGAAGGGGCTG

*M.dunni*  CTGCATATCTACACCTCCCGCCTGTATTTCCACTGGAAGAGGCCCTTCCAGAAGGGGCTG

NIH3T3 CTGCATATCTACACCTCCCGCCTGTATTTCCACTGGAAGAGGCCCTTCCAGAAGGGGCTG

*M.m. domesticus* (prae) CTGCATATCTACACCTCCCGCCTGTATTTCCACTGGAAGAGGCCCTTCCAGAAGGGGCTG

*M.spicilegus*  CCGCATATCTACACCTCCCGCCTGTATTTCCACTGGAAGAGGCCCTTCCAGAAGGGGCTG

*M.setulosus*  CTGCACATCTACACATCCCGCCTGTATTTCCACTGGAAGAGGGCCTTCCAGGAGGGGCTG

*M.shortridgei*  CTGCACATCTACACTTCCCGCCTGTATTTCCACTGGAGGAGGATCTTCCAGAAGGGGCTG

C57BL TGTTCTCTGTGGCAATCAGGGATCCTGGTGGACGTCATGGACCTCCCACAGTTTACTGAC

*M.m. castaneus* TGTTCTCTGTGGCAATCAGGGATCCTGGTGGACGTCATGGACCTCCCACAGTTTACTGAC

*M.dunni*  TGTTCTCTGTGGCAATCAGGGATCCTGGTGGACGTCATGGACCTCCCACAGTTTACTGAC

NIH3T3 TGTTCTCTGTGGCAATCAGGGATCCTGGTGGACGTCATGGACCTCCCACAGTTTACTGAC

*M.m. domesticus* (prae) TGTTCTCTGTGGCAATCAGGGATCCTGGTGGACGTCATGGACCTCCCACAGTTTACTGAC

*M.spicilegus*  TGTTCTCTGTGGCAATCAGGGATCCTGGTGGACGTCATGGACCTCCCACAGTTTACTGAC

*M.setulosus*  TGTTCTCTGTGGCGATCAGGGATCCAGGTGGACGTCATGGACCTCCCACAGTTTACTGAC

*M.shortridgei*  TGTTCTCTGTGGCGATCAGGGATTCAGGTGGACGTCATGGACCTYCCACAGTTTACTGAC

C57BL TGCTGGACAAACTTTGTGAACCCGAAAAGGCCGTTTTGGCCATGGAAAGGATTGGAGATA

*M.m. castaneus*  TGCTGGACAAACTTTGTGAACCCGAAAAGGCCGTTTTGGCCATGGAAAGGATTGGAGATA

*M.dunni*  TGCTGGACAAACTTTGTGAGCCCGAAAAGGCCGTTTTGGCCATGGAAAGGACTGGAGAAA

NIH3T3 TGCTGGACAAACTTTGTGAACCCGAAAAGGCCGTTTTGGCCATGGAAAGGATTGGAGATA

*M.m. domesticus* (prae) TGCTGGACAAACTTTGTGAACCCGAAAAGGCCGTTTTGGCCATGGAAAGGATTGGAGATA

*M.spicilegus*  TGCTGGACAAACTTTGTGAACCCGAAAAGGCCGTTTTGGCCATGGAAAGGATTGGAGATA

*M.setulosus*  TGCTGGACAAACTTTGTGAACCCGCAAAAGCCGTTTAGGCCATGGAAGGGACTGGAGAAA

*M.shortridgei*  TGCTGGACAAACTTTGTGAACCCGCAAAGGCCGTTTAGGCCATGGAAGGGMCTGGAGAAA

C57BL ATCAGCAGGCGCACACAAAGGCGGCTCCGCAGGATCAAGGAGTCCTGGGGTCTGCAAGAT

*M.m. castaneus*  ATCAGCAGGCGCACACAAAGGCGGCTCCACAGGATCAAGGAGTCCTGGGGTCTGCAAGAT

*M.dunni*  ACCAGCAGGCGCACACAAAGGCGGCTCTGCAGGATCAAGGAGTCCCGGGGTCTGCAAGAT

NIH3T3 ATCAGCAGGCGCACACAAAGGCGGCTCCACAGGATCAAGGAGTCCTGGGGTCTGCAAGAT

*M.m. domesticus* (prae) ATCAGCAGGCGCACACAAAGGCGGCTCCACAGGATCAAGGAGTCCTGGGGTCTGCAAGAT

*M.spicilegus*  ATCAGCAGGCGCACACAAAGGCGGCTCGGCAGGATCAAGGAGTCCTGGGGTCTGCAACAT

*M.setulosus*  AACGTCAGGCGCACACAAAGGCGGCTCCGCAGGATCAAGGAGTCCTGGGGCCTGCAAGAT

*M.shortridgei*  AACAGCAGGCACACACAAAGGCGGCTCCGCAGGATCAAGGAGTCCTGGGGCCTGCAAGAT

C57BL TTGGTGAATGAC

*M.m. castaneus*  TTGGTGAATGAC

*M.dunni*  TTGGTGAATGAC

NIH3T3 TTGGTGAATGAC

*M.m. domesticus* (prae) TTGGTGAATGAC

*M.spicilegus*  TTGGTGAATGAC

*M.setulosus*  TTGGTGAATGAC

*M.shortridgei*  TTGGTGAATGAC
