# Supplementary material for: Adaptive Evolution of Mus Apobec3 Includes Retroviral Insertion and Positive Selection at Two Clusters of Residues Flanking the Substrate Groove
Source: PLoS Pathog. 2010 Jul 1;6(7):e1000974. doi: 10.1371/journal.ppat.1000974 (PMC2895647; doi:10.1371/journal.ppat.1000974)
Supplement: Text S2 — Alignment of mA3 Exon 2–4 sequences from mice listed in Table S1. The codons under positive selection by PAML are boxed, with red boxes indicating codons under very strong selection (P>.99). Green fill marks codons that distinguish C57BL and BALB/c. The C57BL mA3 sequence was from GenBank (No. NM_030255). BALB/c exons 3 and 4 were amplified from mRNA and exon 2 from DNA. (0.08 MB DOC) [file ppat.1000974.s006.doc]

C57BL GAAACATTCAAGTTCCACTTTAAGAACCTAGGCTATGCCAAAGGCCGGAAAGATACCTTC

NIH 3T3 GAAACATTCAAATTCCACTTTAAGAACCTACGCTATGCCATAGACCGGAAAGATACCTTC

*M.m. castaneus*-1 GAAACATTCAAGTTCCACTTTAAGAACCTAGGCTATGCCAAAGGCCGGAAAGATACCTTC

*M.m. molossinus* GAAACATTCAAGTTCCACTTTAAGAACCTAGGCTATGCCGAAGGCCGGAAAGATACCTTC

*M.m. musculus*(CzI) GAAACATTCAAGTTCCACTTTAAGGACCTACGCTATGCCAAAGGCCGGAAAGATACCTTC

*M. musculus*(Cal) GAAACATTCAAGTTCCACTTTAAGAACCCACGCTATGCCATAGGCCGGAAAGATACCTTC

NZO GAAACATTCAAATTCCACTTTAAGAACCTACGCTATGCCATAGACCGGAAAGATACCTTC

*M.m. castaneus*-2 GAAACATTCAAATTCCACTTTAAGAACCTACGCTATGCCAAAGGCCGGAAAGATACCTTC

*M.m. domesticus*(prae) GAAACATTCAAATTCCACTTTAAGAACCTACGCTATGCCAAAGGCCGGAAAGATACCTTC

*M.m. domesticus*(JJD) GAAACATTCAAATTCCACTTTAAGAACCTACGCTATGCCATAGACCGGAAAGATACCTTC

*M. spretus* GAAACATTCAAATTCCACTTTAAGAACCTACGCTATGCCATAGACCGGAAAGATACCTTC

*M. macedonicus* GAAACATTCAAATTCCACTTTAAGAACCYACGCTATGCCAAAAACCGGAAAGATACCTTC

*M. spicilegus*-1 GAAACATTCAAATTCCACTTTAAGAACCTACACTATGCCAAAGACCGGAAAGATACCTTC

*M. spicilegus*-2 GAAACATTCAAATTCCACTTCAAGAACCTACGCTATGCCAAAGACCGGAAAGATACCTTC

*M. dunni* GAAACATTCAAGTTCCACTTTGAGAACCTACCCTTTGCCAAAAAACGGAAAGATACCTTC

*M. fragilicauda* GAAACATTCAAGTTCCACTTTAAGAACCTACGCTATGCCATAAACCGGAAAGATACCTTC

*M. caroli* GAAACATTCAAGTTCCACTTTGAGAACCTACGCTATGCCAAAAACCGGAATAATACCTTC

*M. cervicolor* GAAACATTCAATTTCCACTTTAACAACCTATGCTATGCCAAAGGCCGGAACGATACCTTC

*M. cookii* GAAACATTCAATTTCCACTTTAACAACCTATGCTACGCTAAAAACCGGAACGATACCTTC

*M. tenellus* GAAACATTCAAATTCCACTTTAAGAACCTACACTATGCCAAAGACCGGAAAGATACCTTC

*M. setulosus* GAAACATTCAACTTCAACTTTGAGAACCTATGCTATGCCGAAGGCCGAAAAAATACCTTC

*M. minutoides* GAAACATTCAACTTCAACTTTGAGAACCTATGCTATGCCAAAGGCCGGAAAAATACCTTC

*M. saxicola* GAAGACTTCAACTTCCACTTTAAGAACCTACGCTATGCCAAAGGCCGGAGAAATACCTTC

*M. shortridgei* GAAAAATTCGACTTCCACTTTAAGAACCTATGCTATGCCAAAGACCGGAAAGATACCTTC

*M. pahari* GAAAAATTCGACTTCCACTTTAAGAACCTATGCTATGCCAAAGACCGGAAAGATACCTTC

*R. norvegicus* CAAACATTCTACTTTCATTTTAAGAACGTACGCTATGCCTGGGGTCGAAAGAATAACTTC

C57BL TTGTGCTATGAAGTGACTAGAAAGGACTGCGATTCACCCGTCTCCCTTCACCATGGGGTC

NIH 3T3 TTGTGCTATGAAGTGACTAGAAAGGACTGCGATTCACCCGTCTCCCTTCACCATGGGGTC

*M.m. castaneus*-1 TTGTGCTATGAAGTGACTAGAAAGGACTGCGATTCACCCGTCTCCCTTCACCATGGGGTC

*M.m. molossinus* TTGTGCTATGAAGTGACTAGAAAGGACTGCGATTCACCCGTCTCCCTTCACCATGGGGTC

*M.m. musculus*(CzI) TTGTGCTATGAAGTGACTAGAAAGGACTGCGATTCACCCGTCTCCCTTCACCATGGGGTC

*M. musculus*(Cal) TTGTGCTATGAAGTGACTAGAAAGGACTGCGATTCACCCGTCTCCCTTCACCATGGGGTC

NZO TTGTGCTATGAAGTGACTAGAAAGGACTGCGWTTCACCCGTCTCCCTTCACCATGGGGTC

*M.m. castaneus*-2 TTGTGCTATGAAGTGACTAGAAAGGACTGCGATTCACCCGTCTCCCTTCACCATGGGGTC

*M.m. domesticus*(prae) TTGTGCTATGAAGTGACTAGAAAGGACTGCGATTCACCCGTCTCCCTTCACCATGGGGTC

*M.m. domesticus*(JJD) TTGTGCTATGAAGTGACTAGAAAGGACTGCGATTCACCCGTCTCCCTTCACCATGGGGTC

*M. spretus* TTGTGCTATGAAGTGACTAGAAAGGACTGCGATTCACCCGTCTCCCTTCACCATGGGGTC

*M. macedonicus* TTGTGCTATGAAGTGACTAGAAAGGACTGCGATTCACCCGTCTCCCTTCACCATGGGGTC

*M. spicilegus*-1 TTGTGCTATGAAGTGACTAGAAAGGACTGCGATTCACCCGTCTCCCTTCACCATGGGGTC

*M. spicilegus*-2 TTGTGCTATGAAGTGACTAGAAAGGACTGCGATTCACCCGTCTCCCTTCACCATGGGGTC

*M. dunni* TTGTGCTATGAAGTGACTAGAAAGGACTGCGATTCACCCGTCTCCCTTCACCATGGGGTC

*M. fragilicauda* TTGTGCTATGAAGTGACTAGAAAGGACTGCGATTCACCCGTCTCCCTTCACCATGGGGTC

*M. caroli* TTGTGCTATGAAGTGACTAGAAAGGACTGCGATTCACCCGTCTCCCTTTACCATGGGGTC

*M. cervicolor* TTGTGCTATGAAGTGACTAGAAAGGACTGCGATTCACCCGTCTCCCTTCACCATGGGGTC

*M. cookii* TTGTGCTATGAAGTGACTAGAAAGGACTGCGATTCACCCATCTCCCTTCACCATGGGGTC

*M. tenellus* TTGTGCTATGAAGTGACTAGAAAGGACTGCGATTCACCCGTCTCCCTTCACCATGGGGTC

*M. setulosus* TTGTGCTATGAAGTGACTAGAAAGGACTGTGATTCACCCGTTTCCCTTTGCCATGGAGTC

*M. minutoides* TTGTGCTATGAAGTGACTAGAAAGGACTGTGATTCACCCGTTTCCCTTTGCCATGGGGTC

*M. saxicola* CTGTGCTATGAAGTGACTAGAAAGGACTGCAATTCACCCGTCTCCCTTTGCCAYGGGGTC

*M. shortridgei* TTGTGCTATGAAGTGACTAGAAAGGACTGCGATTCACCCAACTCCCTTTGCCATGGGGTC

*M. pahari* TTGTGCTATGAAGTGACTAGAAAGGACTGCGATTCACCCAACTCCCTTTGCCATGGGGTC

*R. norvegicus* TTGTGCTATGAAGTGAATGGGATGGACTGCGCTTTACCTGTCCCCCTTCGCCAAGGGGTC

C57BL TTTAAGAACAAGGACAACATCCACGCTGAAATCTGCTTTTTATACTGGTTCCATGACAAA

NIH 3T3 TTTAAGAACAAGGACAACATCCACGCTGAAATCTGCTTTTTATACTGGTTCCATGACAAA

*M.m. castaneus*-1 TTTAAGAACAAGGACAACATCCACGCTGAAATCTGCTTTTTATACTGGTTCCATGACAAA

*M.m. molossinus* TTTAAGAACAAGGACAACATCCACGCTGAAATCTGCTTTTTATACTGGTTCCATGACAAA

*M.m. musculus*(CzI) TTTAAGAACAAGGACAACATCCACGCTGAAATCTGCTTTTTATACTGGTTCCATGACAAA

*M. musculus*(Cal) TTTAAGAACAAGGACAACATCCACGCTGAAATCTGCTTTTTATACTGGTTCCATGACAAA

NZO TTTAAGAACAAGGACAACATCCACGCTGAAATCTGCTTTTTATACTGGTTCCATGACAAA

*M.m. castaneus*-2 TTTAAGAACAAGGACAACATCCACGCTGAAATCTGCTTTTTATACTGGTTCCATGACAAA

*M.m. domesticus*(prae) TTTAAGAACAAGGACAACATCCACGCTGAAATCTGCTTTTTATACTGGTTCCATGACAAA

*M.m. domesticus*(JJD) TTTAAGAACAAGGACAACATCCACGCTGAAATCTGCTTTTTATACTGGTTCCATGACAAA

*M. spretus* TTTAAGAACAAGGACAACATCCACGCTGAAATCTGCTTTTTATACTGGTTCCATGACAAA

*M. macedonicus* TTTAAGAACAAGGACAACATCCACGCTGAAATCTGCTTTTTATACTGGTTCCATGACAAA

*M. spicilegus*-1 TTTAAGAACAAGGACAACATCCACGCTGAAATCTGCTTTTTATACTGGTTCCATGACAAA

*M. spicilegus*-2 TTTAAGAACAAGGACAACATCCACGCTGAAATCTGCTTTTTATACTGGTTCCATGACAAA

*M. dunni* TTTAAGAACAAGGACAACATCCACGCTGAAATCTGCTTTTTATACTGGTTCCATGACAAA

*M. fragilicauda* TTTAAGAACAAGGACAACATCCACGCTGAAATCTGCTTTTTATACTGGTTCCATGACAAA

*M. caroli* TTTACGAACAAGGACGACATCCACGCTGAAATCTGCTTTTTATACTGGTTCCATGACAAA

*M. cervicolor* TTTAAGAWCAAGGACAACATCCACGCTGAAATCTGCTTTTTATACTGGTTCCATGACAAA

*M. cookii* TTTAAGAACAAGGACAACATCCATGCTGAAATCTGCTTTTTATACTGGTTCCATGACAAA

*M. tenellus* TTTAAGAACAAGGGCAGCATTCATGCCGAAATCTGCTTTTTATACTGGTTCCATAACAAA

*M. setulosus* TTTAAGAACAAGGGCAGCATCCACGCTGAAATCTGCTTTTTATACTGGTTCCATGACAAA

*M. minutoides* TTTAAGAACAAGGGCAGCATCCACGCTGAAATCTGCTTTTTATACTGGTTCCATGACAAA

*M. saxicola* TTTGAGAACAAGGGCATCATCCACGCTGAAATCTGCTTTTTATACTGGTTTCATGACAAA

*M. shortridgei* TTTAAGAACAAGGGCAACACCCACGCTGAAATCTGCTTTTTATACTGGTTCCATGACAAA

*M. pahari* TTTAAGAACAAGGGCAACACCCACGCTGAAATCTGCTTTTTATACTGGTTCCATGACAAA

*R. norvegicus* TTCAGGAAACAGGGCCACATCCACGCCGAACTCTGCTTCATATACTGGTTCCACGACAAA

C57BL GTACTGAAAGTGCTGTCTCCGAGAGAAGAGTTCAAGATCACCTGGTATATGTCCTGGAGC

NIH 3T3 GTACTGAAAGTGCTGTCTCCGAGAGAAGAGTTCAAGATCACCTGGTATATGTCCTGGAGC

*M.m. castaneus*-1 GTACTGAAAGTGCTGTCTCCGAGAGAAGAGTTCAAGATCACCTGGTATATGTCCTGGAGC

*M.m. molossinus* GTACTGAAAGTGCTGTCTCCGAGAGAAGAGTTCAAGATCACCTGGTATATGTCCTGGAGC

*M.m. musculus*(CzI) GTACTGAAAGTGCTGTCTCCGAGAGAAGAGTTCAAGATCACCTGGTATATGTCCTGGAGC

*M. musculus*(Cal) GTACTGAAAGTGCTGTCCCCGAGAGAAGAGTTCAAGATCACCTGGTATATGTCCTGGAGC

NZO GTACTGAAAGTGCTGTCTCCGAGAGAAGAGTTCAAGATCACCTGGTATATGTCCTGGAGC

*M.m. castaneus*-2 GTACTGAAAGTGCTGTCTCCGAGAGAAGAGTTCAAGATCACCTGGTATATGTCCTGGAGC

*M.m. domesticus*(prae) GTACTGAAAGTGCTGTCTCCGAGAGAAGAGTTCAAGATCACCTGGTATATGTCCTGGAGC

*M.m. domesticus*(JJD) GTACTGAAAGTGCTGTCTCCGAGAGAAGAGTTCAAGATCACCTGGTATATGTCCTGGAGC

*M. spretus* GTACTGAAAGTGCTGTCTCCGAGAGAAGAGTTCAAGATCACCTGGTATATGTCCTGGAGC

*M. macedonicus* GTACTGAAAGTGCTGTCTCTGAGAGAAGAGTTCAAGATCACCTGGTATATGTCCTGGAGC

*M. spicilegus*-1 GTACTGAAAGTGCTGTCTCCGAGAGAAGAGTTCAAGATCACCTGGTATATGTCCTGGAGC

*M. spicilegus*-2 GTATTGAAAGTGCTGTCTCCGAGAGAAGAGTTCAAGATCACCTGGTATATGTCCTGGAGC

*M. dunni* GTACTGAAAGTGCTATCTCCGAGGGAAGAGTTCAAGATCACCTGGTATATGTCCTGGAGC

*M. fragilicauda* GTACTGAAAGTGCTGTCTCCGAGGGAAGAGTTCAAGGTCACCTGGTATATGTCCTGGAGC

*M. caroli* GTACTGAAAGTGCTGTCTCCGAGGGAAGAGTTCATGGTCACCTGGTATATGTCCTGGAGC

*M. cervicolor* GTACTGAAAGTKCTGTCTCCGAGGGAAGAGTTCATGRTCACCTGGTATATGTCCTGGAGC

*M. cookii* GTACTGAAAGTGCTGTCTCCGAGGGAAGAGTTCATGGTCACCTGGTATATGTCCTGGAGC

*M. tenellus* GTACTTAGAGTGCTGTCTCCGAGGGAAGAGTTCAAGGTCACCTGGTATACGTCCTGGAGC

*M. setulosus* GTACTGAAAGTGCTGACTCAGAGGGAAGGGTTCAAGGTCACCTGGTATATGTCCTGGAGC

*M. minutoides* GTACTGAAAGTGCTGACTCCGAGGGAAGAGTTCAAGGTCACCTGGTATATGTCCTGGAGC

*M. saxicola* GTACTGAAAGTGCTGTCTCCGAGGGAAGAGTTCAAGGTCACCTGGTATATGTCCTGGAGC

*M. shortridgei* GTACTAAAAGTGCTGTCTCTGAGGGAAGAGTTCAAGGTCACCTGGTACATGTCCTGGAGC

*M. pahari* GTACTAAAAGTGCTGTCTCTGAGGGAAGAGTTCAAGGTCACCTGGTACATGTCCTGGAGC

*R. norvegicus* GTCCTGAGAGTGCTGTCCCCGATGGAAGAGTTCAAGGTCACGTGGTACATGTCCTGGAGC

C57BL CCCTGTTTCGAATGTGCAGAGCAGATAGTAAGGTTCCTGGCTACACACCACAACCTGAGC

NIH 3T3 CCCTGTTTCGAATGTGCAGAGCAGGTACTAAGGTTCCTGGCTACACACCACAACCTGAGC

*M.m. castaneus*-1 CCCTGTTTCGAATGTGCAGAGCAGATAGTAAGGTTCCTGGCTACACACCACAACCTGAGC

*M.m. molossinus* CCCTGTTTCGAATGTGCAGAGCAGATAGTAAGGTTCCTGGCTACACACCACAACCTGAGC

*M.m. musculus(CzI)* CCCTGTTTCGAATGTGCAGAGCAGATAGTAAGGTTCCTGGCTACACACCACAACCTGAGC

*M. musculus*(Cal) CCCTGTTTCGAATGTGCAGAGCAGATAGTAAGGTTCCTGGCTACACACCACAACCTGAGC

NZO CCCTGTTTCGAATGTGCAGAGCAGATAGTAAGGTTCCTGGCTACACACCACAACCTGAGC

*M.m. castaneus*-2 CCCTGTTTCGAATGTGCAGAGCAGGTACTAAGGTTCCTGGCTACACACCACAACCTGAGC

*M.m. domesticus*(prae) CCCTGTTTCGAATGTGCAGAGCAGGTACTAAGGTTCCTGGCTACACACCACAACCTGAGC

*M.m. domesticus*(JJD) CCCTGTTTCGAATGTGCAGAGCAGGTACTAAGGTTCCTGGCTACACACCACAACCTGAGC

*M. spretus* CCCTGTTTCGAATGTGCAGAGCAGGTACTAAGGTTCCTGGCTACACACCACAACCTGAGC

*M. macedonicus* CCCTGTTTCGAATGTGCAGAGCAGGTAGTAAGGTTCCTGGCTACACACCACAACCTGAGC

*M. spicilegus*-1 CCCTGTTTCGAATGTGCAGAGCAGGTAGTAAGGTTCCTGGCTACACACCACAACCTGAGC

*M. spicilegus*-2 CCCTGTTTCGAATGTGCAGAGCAGGTAGTAAGGTTCCTGGCTACACACCACAACCTGAGC

*M. dunni* CCCTGTTTCGAATGTGCAGAGCAGGTAGTAAGGTTCCTGGCCACACACCACAACCTGAGC

*M. fragilicauda* CCCTGTTTCGAATGTGCAGAGCAGGTAGTAAGGTTCCTGGCCACACACCACAACCTGAGC

*M. caroli* CCCTGTTTCAAATGTGCAGAGCAGGTAGTAAGGTTCCTGGCCACACACCACAACCTGAGC

*M. cervicolor* CCCTGTTTCGAATGTGCAGAGCAGGTAGTAAGGTTCTTGGCCACACACCACAACCTGAGC

*M. cookii* CCCTGTTTCGAATGTGCAGAGCAGGTAGTAAGGTTCCTGGCCACACACCACAACGTGAGC

*M. tenellus* CCCTGTTTCAAATGTGCAGAGCAGGTAGCCAGGTTTCTGGCCACACACCACAACCTGAGC

*M. setulosus* CCCTGTTTTGAATGTGCAGAGCAGGTAGTCAGGTTCCTGGCCACACACCACAACCTGAAC

*M. minutoides* CCCTGTTTTGAATGTGCAGAGCAGGTAGTCAGGTTCCTGGCCACACACCACAACCTGAAC

*M. saxicola* CCCTGTTTCAAATGTGCAGAACAGGTAGTCAGGTTCCTGGCCACACACCACAACCTGAGC

*M. shortridgei* CCCTGCTTCGAATGCGCAGAGCAGGTAGCCAGGTTCCTGGCCACACACCACAACCTGAGC

*M. pahari* CCCTGCTTCGAATGCGCAGAGCAGGTAGCCAGGTTCCTGGCCACACACCACAACCTGAGC

*R. norvegicus* CCCTGCAGCAAGTGCGCGGAGCAGGTAGCCAGGTTCCTGGCCGCACACCGCAACCTAAGC

C57BL CTGGACATCTTCAGCTCCCGCCTC---TACAACGTACAGGACCCAGAAACCCAGCAGAAT

NIH 3T3 CTGGACATCTTCAGCTCCCGCCTC---TACAACATACGGGACCCAGAAAACCAGCAGAAT

*M.m. castaneus*-1 CTGGACATCTTCAGCTCCCGCCTC---TACAACGTACAGGACCCAGAAACCCAGCAGAAT

*M.m. molossinus* CTGGACATCTTCAGCTCCCGCCTC---TACAACGTACGGGACCCAGAAACCCAGCAGAAT

*M.m. musculus*(CzI) CTGGACATCTTCAGCTCCCGCCTC---TACAACGTACGGGTCCCAGAAACCCAGCAGAAT

*M. musculus*(Cal) CTGGACATCTTCAGCTCCCGCCTC---TACAACATAGGGGTCCCAGAAACCCAACAGAAT

NZO CTGGACATCTTCAGCTCCCGCCTC---TACAACGTACAGGACCCAGAAACCCAGCAGAAT

*M.m. castaneus*-2 CTGGACATCTTCAGCTCCCGCCTC---TACAACATACGGGACCCAGAAAACCAGCAGAAT

*M.m. domesticus*(prae) CTGGACATCTTCAGCTCCCGCCTC---TACAACATACGGGACCCAGAAAACCAGCAGAAT

*M.m. domesticus*(JJD) CTGGACATCTTCAGCTCCCGCCTC---TACAACATACGGGACCCAGAAAACCAGCAGAAT

*M. spretus* CTGGACATCTTCAGCTCCCGCCTC---TACAACATACGGGACCCAGAAAACCAGCAGAAT

*M. macedonicus* CTGGACATCTTCAGCTCCCGCCTC---TACAAYATACGGGACCCAAAAACCCAGCAGAAT

*M. spicilegus*-1 CTGGACATCTTCATCTCCCGCCTC---TACAACATATTGGACCCAAAAACCCAGCAGAAT

*M. spicilegus*-2 CTGGACATCTTCATCTCCCGCCTC---TACAACATATGGGACCCAAAAACCCAGCAGAAT

*M. dunni* CTGGACATCTTCTTCTCCCGCCTC---TACAACATAAGGAACCCAGAAAACCAGCAGAAT

*M. fragilicauda* CTGGACATCTTCAGCTCCCGCCTC---TACAACGTATGGAACCCAGAAACCCAGCAGAAT

*M. caroli* CTGGACATCTTCAGCTCCCGCCTC---TACAACGTATCGAAACCAGAAACCCAGCAGAAT

*M. cervicolor* CTGGACATCTTCTGCTCCCGCCTC---TACAACGTATCGATCCCAGAAACCCAGCAGAAT

*M. cookii* CTGGACATCTTCAGCTCCCGCCTC---TACAACGTATCGATCCCAGAAACCCAGCAGAAT

*M. tenellus* CTGGTCATCTTCAGCGCCCGCCTC---TACAAAGTATGGGACCCGGGCACCCAGCAGAAA

*M. setulosus* CTGACCATCTTCAGCTCCCGCCTC---TACAATGTATCAGACCCGGACACCCAGCAGAAA

*M. minutoides* CTGACCATCTTCAGCTCCCGCCTC---TACAATGTATCAGACCCGGACACCCAGCAGAAA

*M. saxicola* CTGGCCATCTTTATCTCCCGCCTC---TATGACATATGGAACACCGACACCCAGCAGAAA

*M. shortridgei* CTGGCCATCTTCAGCTCCCGCCTC---TACAACATAAGGGACCCGAAGACCGGGCAGGGA

*M. pahari* CTGGCCATCTTCAGCTCCCGCCTC---TACAACATAAGGGACCCGAAGACCGGGCAGGGA

*R. norvegicus* CTGGCCATCTTCAGCTCCCGCTTGTACTACTACTTAAGGAACCCGAACTACCAGCAGAAG

C57BL CTTTGCAGGCTGGTTCAGGAAGGAGCCCAGGTGGCTGCCATGGACCTATACGAATTTAAA

NIH 3T3 CTTTGCAGGCTGGTTCAGGAAGGAGCCCAGGTGGCTGCCATGGACCTATACGAATTTAAA

*M.m. castaneus*-1 CTTTGCAGGCTGGTTCAGGAAGGAGCCCAGGTGGCTGCCATGGACCTATACGAATTTAAA

*M.m. molossinus* CTTTGCAGGCTGGTTCAGGAAGGAGCCCAGGTGGCTGCCATGGACCTATACGAATTTAAA

*M.m. musculus*(CzI) CTTTGCAGGCTGGTTCAGGAAGGAGCCCAGGTGGCTGCCATGGACCTATACGAATTTAAA

*M. musculus*(Cal) CTTTGCAGGCTGGTTCAGGAAGGAGCCCAGGTGGCTGCCATGGACCTATACGAATTTAAA

NZO CTTTGCAGGCTGGTTCAGGAAGGAGCCCAGGTGGCTGCCATGGACCTATACGAATTTAAA

*M.m. castaneus*-2 CTTTGCAGGCTGGTTCAGGAAGGAGCCCAGGTGGCTGCCATGGACCTATACGAATTTAAA

*M.m. domesticus*(prae) CTTTGCAGGCTGGTTCAGGAAGGAGCCCAGGTGGCTGCCATGGACCTATACGAATTTAAA

*M.m. domesticus*(JJD) CTTTGCAGGCTGGTTCAGGAAGGAGCCCAGGTGGCTGCCATGGACCTATACGAATTTAAA

*M. spretus* CTTTGCAGGCTGGTTCAGGAAGGAGCCCAGGTGGCTGCCATGGACCTATACGAATTTAAA

*M. macedonicus* CTTTGCAGGCTGGTTCAGGAAGGAGCCCAGGTGGCTGCCATGGACCTATACGAATTTAAA

*M. spicilegus*-1 CTTTGCAGGCTGGTTCAGGAAGGAGCCCAGGTGGCTGCCATGGACCTATACGAATTTAAA

*M. spicilegus*-2 CTTTGCAGGCTGGTTCAGGAAGGAGCCCAGGTGGCTGCCATGGACCTATACGAATTTAAA

*M. dunni* CTTTGCAGGCTGGTTCTGGAAGGAGCCCAGGTGGCTGCCATGGACCTATACGAATTTGAA

*M. fragilicauda* CTTTGCAGGCTGGTTCTGGAAGGAGCCCAGGTGGCTGCCATGGACCTATACGATTTTAAA

*M. caroli* CTTTGCAGGCTGGTTCTGGAAGGAGCCCAGGTGGCTGCCATGGACCTATACGAATTTAAA

*M. cervicolor* CTTTGCAGGCTGGTTCAGGAAGGAGCCCRGGTGGCTGTCATGGACCTAGATGAATTTGAA

*M. cookii* CTTTGCAGGCTGGTTCAGGAAGGAGCCCAGGTGGCTGTCATGGGCCTAGACGAATTTGAA

*M. tenellus* CTTTGCAGGCTGGTTCAGGAAGGAGCCCAGGTGGCTGCCATGGACCTATTCGAATTTAAA

*M. setulosus* CTTTGCAGGCTGGTTCAGGAAGGAGCCCAGGTGGCTGTCATGGACCTATCCGAATTTAAA

*M. minutoides* CTTTGCAGGCTGGTTCAGGAAGGAGCCCAGGTGGCTGTCATGGACCTATCCGAATTTAAA

*M. saxicola* CTTTGCAGGCTGGTTCAGGAAGGAGCCCAAGTGGCTGCCATGGACCTACCCGAATTTAAA

*M. shortridgei* CTTTGCAGGCTGGTTCAGGAAGGAGCCCAGGTGGCTGCCATGGGCCTACCCGAATTTGAA

*M. pahari* CTTTGCAGGCTGGTTCAGGAAGGAGCCCAGGTGGCTGCCATGGGCCTACCCGAATTTGAA

*R. norvegicus* CTCTGCAGGCTGATTCAGGAAGGAGTCCACGTGGCTGCCATGGACCTACCAGAATTTAAA

C57BL AAGTGTTGGAAGAAGTTTGTGGACAATGGTGGCAGGCGATTCAGGCCTTGGAAAAGACTG

NIH 3T3 AAGTGTTGGAAGAAGTTTGTGGACAATGGCGGCAGGCGATTCAGGCCTTGGAAAAAACTG

*M.m. castaneus*-1 AAGTGTTGGAAGAAGTTTGTGGACAATGGTGGCAGGCGATTCAGGCCTTGGAAAAGACTG

*M.m. molossinus* AAGTGTTGGAAGAAGTTTGTGGACAATGGTGGCAGGCGATTCAGGCCTTGGAAAAGACTG

*M.m. musculus*(CzI) AAGTGTTGGAAGAAGTTTGTGGACAATGGTGGCAGGCGATTCAGGCCTTGGAAAAGACTG

*M. musculus*(Cal) AAGTGTTGGAAGAAGTTTGTGGACAACGGTGGCAGGCGATTCAGGCCTTGGAAAAGACTG

NZO AAGTGTTGGAAGAAGTTTGTGGACAATGGTGGCAGGCGATTCAGGCCTTGGAAAAGACTG

*M.m. castaneus*-2 AAGTGTTGGAAGAAGTTTGTGGACAATGGCGGCAGGCGATTCAGGCCTTGGAAAAAACTG

*M.m. domesticus*(prae) AAGTGTTGGAAGAAGTTTGTGGACAATGGCGGCAGGCGATTCAGGCCTTGGAAAAAACTG

*M.m. domesticus*(JJD) AAGTGTTGGAAGAAGTTTGTGGACAATGGCGGCAGGCGATTCAGGCCTTGGAAAAAACTG

*M. spretus* AAGTGTTGGAAGAAGTTTGTGGACAATGGCGGCAGGCGATTCAGGCCTTGGAAAAAACTG

*M. macedonicus* AAGTGTTGGAAGAAGTTTGTGGACAACGGCGGCAGGCGATTCAGGCCTTGGAAAAGACTG

*M. spicilegus*-1 AAGTGTTGGAAGAAGTTTGTGGACAACGGCGGCAGGCGATTCAGGCCTTGGAAAAGACTG

*M. spicilegus*-2 AAGTGTTGGAAGAAGTTTGTGGACAACGGCGGCAGGCGATTCAGGCCTTGGAAAAGACTG

*M. dunni* GAGTGTTGGAAGAAGTTTGTGGACAATGGCGGCAGACGATTCAGGCCTTGGAAAAGACTG

*M. fragilicauda* AAGTGTTGGAAGAAGTTTGTGGACAATGGCGGCAGGCGATTCAGGCCTTGGAAAAGACTG

*M. caroli* AAGTGTTGGAAGAAGTTTGTGGACAATGACGGCAGGCGATTCAGGCCTTGGGAAAGCCTG

*M. cervicolor* AAGTGTTGGAAGAAGTTTGTGGACAATGGYGGCAGGCCATTCAGGCCTTGGAAAAATCTG

*M. cookii* AAGTGTTGGAAGAAGTTTGTGGACAATGGCGGCAGGCGATTCAGGCCTTGGAAAAGTCTG

*M. tenellus* AAGTGTTGGGAGAAGTTTGTGGACAATCATGGCCAGCGATTCAGGCCTTGGAAGAGACTG

*M. setulosus* AAGTGTTGGGAGAAGTTTGTGGACAATGACGGCCAGCAATTCAGGCCTTGGAAGAGACTG

*M. minutoides* AAGTGTTGGGAGAAGTTTGTGGACAATGACGGCCAGCAATTCAGGCCTTGGAAGAGACTG

*M. saxicola* AAGTGTTGGAAGAAGTTTGTGGACAATGACGGCCAGCGATTCAGGCCTTGGAAGAGACTA

*M. shortridgei* AAGTGTTGGAAGAAGTTTGTGGACAATGACGGCCAGCCATTCATGCCTTGGAAGAGACTG

*M. pahari* AAGTGTTGGAAGAAGTTTGTGGACAATGACGGCCAGCCATTCATGCCTTGGAAGAGACTG

*R. norvegicus* AAGTGTTGGAACAAGTTTGTGGACAATGACGGCCAACCATTCAGGCCTTGGATGAGACTG

C57BL CTTACAAATTTTAGATACCAGGATTCTAAGCTTCAGGAGATTCTG

NIH 3T3 CTTACAAATTTTAGATACCAGGATTCTAAGCTTCAGGAGATTCTG

*M.m. castaneus*-1 CTTACAAATTTTAGATACCAGGATTCTAAGCTTCAGGAGATTCTG

*M.m. molossinus* CTTACAAATTTTAGATACCAGGATTCTAAGCTTCAGGAGATTCTG

*M.m. musculus(CzI)* CTTACAAATTTTAGATACCAGGATTCTAAGCTTCAGGAGATTCTG

*M. musculus*(Cal) CTTGCAAATTTTAGATACCAGGATTCTAAGCTTCAGGAGATTCTG

NZO CTTACAAATTTTAGATACCAGGATTCTAAGCTTCAGGAGATTCTG

*M.m. castaneus*-2 CTTACAAATTTTAGATACCAGGATTCTAAGCTTCAGGAGATTCTG

*M.m. domesticus*(prae) CTTACAAATTTTAGATACCAGGATTCTAAGCTTCAGGAGATTCTG

*M.m. domesticus*(JJD) CTTACAAATTTTAGATACCAGGATTCTAAGCTTCAGGAGATTCTG

*M. spretus* CTTACAAATTTTAGATACCAGGATTCTAAGCTTCAGGAGATTCTG

*M. macedonicus* CTTACAAATTTTAGATACCAGGATTCTAAGCTTCAGGAGATTCTG

*M. spicilegus*-1 CTTACAAATTTTAGATACCAGGATTCTAAGCTTCAGGAGATTCTG

*M. spicilegus*-2 CTTACAAATTTTAGATACCAGGATTCTAAGCTTCAGGAGATTCTG

*M. dunni* CTTACAAATTTTAGATACCAGGATTCTAAGCTTCAGGAGATTCTG

*M. fragilicauda* CTTACAAATTTTAGATACCAGGATTCTAAGCTTCAGGAGATTCTG

*M. caroli* CTTACAAATTTTAGATACCAGGATTCTAAGCTTCAGGAGATTCTG

*M. cervicolor* CTTACAAATTTTAGATACCATGATTCTAAGCTTCAGGAGATTCTG

*M. cookii* CTTACGAATTTTAGATACCAGGATTCTAAGCTTCAGGAGATTCTG

*M. tenellus* CGTACAAATTTTAGATACCAGAATTCTAAGCTTCAGGAGATTCTG

*M. setulosus* CATACCAATTTTAGATATCAGAATTCTAAGCTTCAGGAGATTCTG

*M. minutoides* CGTACCAATTTTAGATATCAGAATTCTAAGCTTCAGGAGATTCTG

*M. saxicola* CGTACACATTTTAGATACCAGAATTCTAAGCTTCAGGAGATTCTG

*M. shortridgei* TGTACAAATTTTAGATACCAGAATTCTAAGCTTCAGGAGATTCTG

*M. pahari* TGTACAAATTTTAGATACCAGAATTCTAAGCTTCAGGAGATTCTG

*R. norvegicus* AGAATAAATTTTAGTTTCTATGATTGCAAGCTTCAGGAGATTTTC
